# A novel AA14 LPMO from *Talaromyces rugulosus* with bifunctional cellulolytic/hemicellulolytic activity boosted cellulose hydrolysis

**DOI:** 10.1186/s13068-024-02474-9

**Published:** 2024-02-23

**Authors:** Kaixiang Chen, Xu Zhao, Peiyu Zhang, Liangkun Long, Shaojun Ding

**Affiliations:** The Co-Innovation Center of Efficient Processing and Utilization of Forest Resources, Jiangsu Key Lab for the Chemistry & Utilization of Agricultural and Forest Biomass, College of Chemical Engineering, Nanjing Forestry University, Nanjing, 210037 Jiangsu China

**Keywords:** Auxiliary activity family 14, Lytic polysaccharide monooxygenase, *Talaromyces rugulosus*, Cellulose, Xylan, Xyloglucan

## Abstract

**Background:**

The recently discovered *Pc*AA14A and B from white-rot basidiomycete *Pycnoporus coccineus* enriched our understanding of the oxidative degradation of xylan in fungi, however, the unusual mode of action of AA14 LPMOs has sparked controversy. The substrate specificity and functionality of AA14 LPMOs still remain enigmatic and need further investigation.

**Results:**

In this study, a novel AA14 LPMO was characterized from the ascomycete *Talaromyces rugulosus*. *Tr*AA14A has a broad substrate specificity with strong oxidative activity on pure amorphous cellulose and xyloglucan. It could simultaneously oxidize cellulose, xylan and xyloglucan in natural hemi/cellulosic substrate such as fibrillated eucalyptus pulp, and released native and oxidized cello-oligosaccharides, xylo-oligosaccharides and xyloglucan oligosaccharides from this substrate, but its cellulolytic/hemicellulolytic activity became weaker as the contents of xylan increase in the alkaline-extracted hemi/cellulosic substrates. The dual cellulolytic/hemicellulolytic activity enables *Tr*AA14A to possess a profound boosting effect on cellulose hydrolysis by cellulolytic enzymes. Structure modelling of *Tr*AA14A revealed that it exhibits a relatively flat active-site surface similar to the active-site surfaces in AA9 LPMOs but quite distinct from *Pc*AA14B, despite *Tr*AA14A is strongly clustered together with AA14 LPMOs. Remarkable difference in electrostatic potentials of L2 and L3 surfaces was also observed among TrAA14A, *Pc*AA14B and *Nc*LPMO9F. We speculated that the unique feature in substrate-binding surface might contribute to the cellulolytic/hemicellulolytic activity of *Tr*AA14A.

**Conclusions:**

The extensive cellulolytic/hemicellulolytic activity on natural hemi/cellulosic substrate indicated that *Tr*AA14A from ascomycete is distinctively different from previously characterized xylan-active AA9 or AA14 LPMOs. It may play as a bifunctional enzyme to decompose some specific network structures formed between cellulose and hemicellulose in the plant cell walls. Our findings shed new insights into the novel substrate specificities and biological functionalities of AA14 LPMOs, and will contribute to developing novel bifunctional LPMOs as the booster in commercial cellulase cocktails to efficiently break down the hemicellulose-cellulose matrix in lignocellulose.

**Supplementary Information:**

The online version contains supplementary material available at 10.1186/s13068-024-02474-9.

## Background

Lytic polysaccharide monooxygenases (LPMOs) are a group of redox-active enzymes that catalyze the oxidative cleavage of glycosidic bonds of recalcitrant polysaccharides, such as cellulose and chitin [1–3]. The boosting effect of these monocopper enzymes on the enzymatic hydrolysis of recalcitrant polysaccharides has extensively promoted fundamental and applied research, leading to significant progress in understanding LPMOs and harnessing the potential of these enzymes in industrial biomass conversion [4–10]. AA9 LPMOs, originally designated as glycoside hydrolase family 61, was firstly identified from fungi [2]. To date, the number of fungal LPMO families with various substrate specificities and biological functionalities has been expanded to five AA families (AA9, AA11, AA13, AA14, and AA16) in the Carbohydrate Active enZymes (CAZy, www.cazy.org) database. Fungal LPMOs from AA9, AA11, AA13, and AA16 exhibit well-defined substrate specificity towards cellulose, chitin, starch, and cellulose, respectively [2, 11–13]. In 2014, Agger et al. [14] found that *Nc*LPMO9C from *Neurospora crassa*, could degrade various hemicelluloses, in particular xyloglucan. In 2015, Frommhagen et al. [15] discovered the first AA9 LPMO (*Mt*LPMO9A) from *Myceliophthora thermophila* C1, which shows oxidative cleavage of xylan in addition to cellulose and xyloglucan, and acts in synergism with endoglucanase I. Since hemicelluloses such as xylan and xyloglucan are closely associated with cellulose, the oxidative cleavage of the hemicellulose-coated cellulose regions by hemicellulolytic activity of AA9 LPMOs could be important for loosening the rigid plant polysaccharide matrix in plant biomass, enabling an increased accessibility for hydrolytic enzymes [14, 15]. However, the xylan-activity of most identified AA9 LPMOs was not highly specific, and was usually in lower level compared to their activity on cellulose.

The recently discovered AA14 LPMOs from white-rot basidiomycete *Pycnoporus coccineus* displayed a unique feature that did not show any activity on xylan polymers or underlying cellulose fibrils in a wide range of hemi/cellulosic substrates in the presence of ascorbic acid [16]. ESI–MS analysis revealed that C1-oxidized xylo-oligosaccharides could only be generated from birchwood cellulosic fibers in synergy with GH11 xylanase. Further study demonstrated that *Pc*AA14B also showed a strong synergistic interaction with *Tt*Xyn30A in degrading the recalcitrant part of xylan of birchwood cellulosic fibers [17]. However, the effect of the *Pc*AA14B–*Tt*Xyn30A synergism was more prominent on substrates with low hemicellulose content, in which xylan is in close proximity to the underlying cellulose fibers, while on substrates with high xylan content, the synergism was entirely absent. Both AA9 and AA14 LPMOs are widely distributed among white-rot, brown-rot basidiomycetes, and ascomycetes [16]. A wood-decaying ascomycete or basidiomycete fungus typically contains multiple AA9 LPMO-encoding genes (up to 30), but only has 1–4 AA14 LPMO-encoding genes in their genomes [16, 18, 19]. So far, only two AA14 LPMOs (*Pc*AA14A and *Pc*AA14B) have been characterized from white-rot basidiomycete *P. coccineus*. However, the unusual mode of action of AA14 LPMOs has sparked controversy. Recently, Tuveng et al. [20] published the revisiting work on the *Pc*AA14A from *P. coccineus*. Unexpectedly, they could not detect the previously proposed oxidative activity on cellulose-associated xylans, and the synergistic effect of *Pc*AA14A with xylanase, although *Pc*AA14A has oxidase and peroxidase activities that are common for LPMOs. Intriguingly, AA14 sequences are particularly enriched in the intrinsically disordered C-terminal regions (dCTRs) (57%), i.e., both *Pc*oAA14A and *Pc*oAA14B harbor a long dCTR of 144- and 136-amino acid residues, respectively [21]. Therefore, the substrate specificity and functionality of these new AA family enzymes still remain enigmatic and need further investigation.

*Talaromyces rugulosus* is a filamentous ascomycete fungus in the family of *Trichocomaceae*. It is rich in carbohydrate-active enzymes, especially abundant in glycoside hydrolase family enzymes, but it only contains one AA9 LPMO- (GenBank No. QKX62378.1) and two AA14 LPMO-encoding genes (GenBank No. QKX59718.1 and QKX64748.1) [22]. The difference in the number and type of carbohydrate-active enzyme-encoding genes in this ascomycete fungal genome from other cellulolytic fungi attracted our attention to the role of AA14 LPMO. We hypothesized that AA14 LPMO from this ascomycete fungus might exhibit different substrate specificities and functionalities in hemi/cellulose degradation compared to basidiomycete fungus. In the present work, one AA14 LPMO (*Tr*AA14A) from *T. rugulosus* W13939 was characterized. Our work demonstrated that *Tr*AA14A displayed distinctive cellulose- and xyloglucan-activity on pure amorphous cellulose and xyloglucan at C1-carbon, and probably also at C4-carbon. It could simultaneously oxidize cellulose, xylan and xyloglucan in natural hemi/cellulosic substrate such as fibrillated eucalyptus pulp. Furthermore, *Tr*AA14A boosted the cellulose hydrolysis by glycoside hydrolases (GHs). This discovery significantly expanded our understanding of the functional diversity of AA14 LPMOs between basidiomycetes and ascomycetes. The unique characteristics of the novel *Tr*AA14A imply that it may function as a bifunctional enzyme to overcome the recalcitrance of lignocellulose by *T. rugulosus* in nature and also be significant for industrial applications in the biorefinery field.

## Results

### Phylogenetic analysis and sequence alignment of AA14 LPMOs

**Fig. 1:**
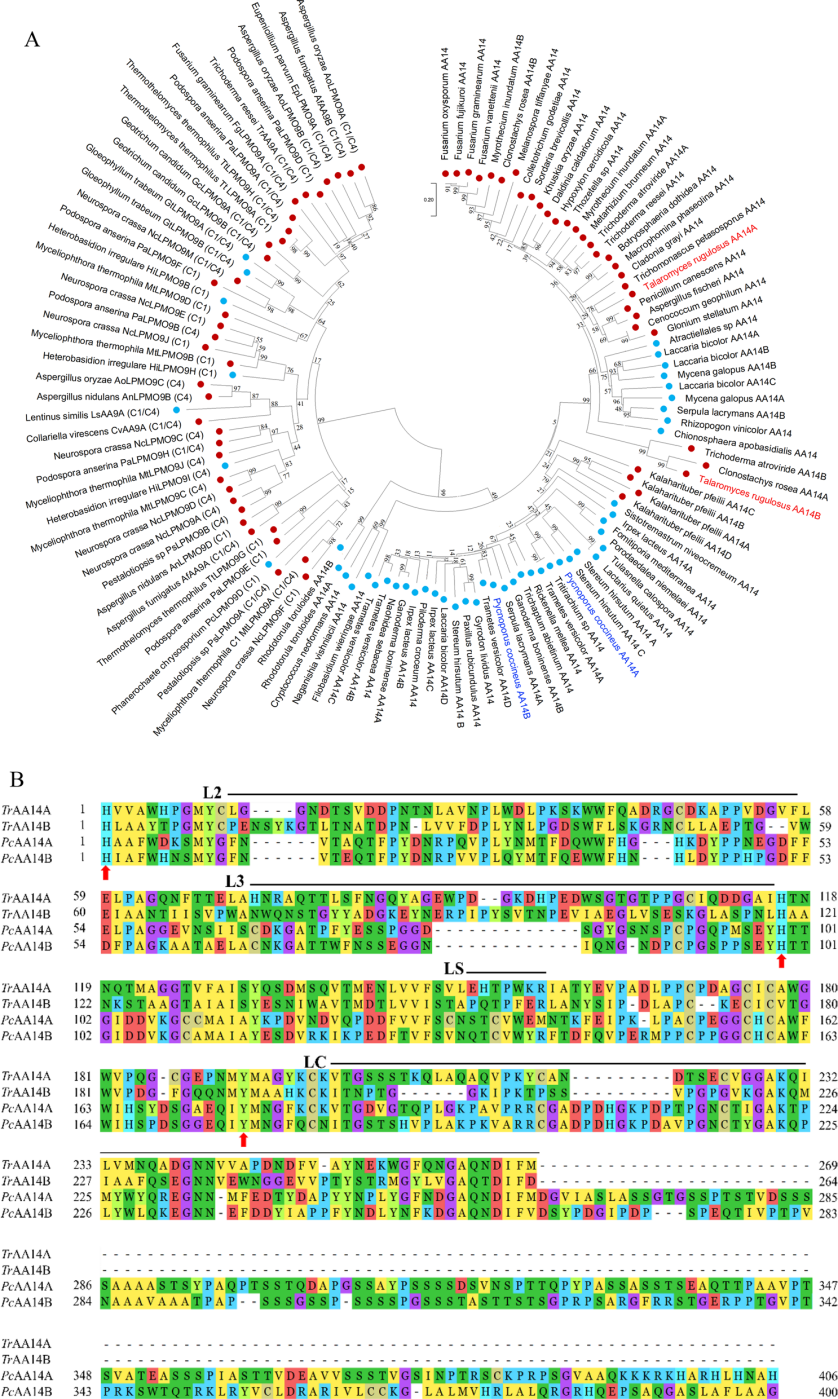
Phylogenetic analysis and sequence alignment of AA14 LPMOs. **A** Phylogenetic tree of putative AA14 LPMOs and characterized AA9 LPMOs. The sequences encoding signal peptide, CBM1 and the dCTR based on the prediction using MobiDB-lite 5.0 (https://mobidb.bio.unipd.it/) in selected genes were deleted, and only the catalytic domain was selected for phylogenetic analysis. Blue dot indicates proteins from Basidiomycota, while red dot indicates proteins from Ascomycota **B** Sequence alignment of *Tr*AA14A and 14B with *Pc*AA14A and 14B. The amino acid residues forming the His brace are indicated as the solid red arrow

Based on the neighbor-joining phylogenetic tree, two AA14 LPMOs from the *T. rugulosus* W13939 are very distant from AA9 LPMOs, but strongly cluster together with AA14 LPMOs from basidiomycete and ascomycete fungi (Fig. 1A). Thus, *Tr*AA14A and *Tr*AA14B belong to the AA14 family member. Unlike *Pc*AA14A and *Pc*AA14B, both *Tr*AA14A and *Tr*AA14B lack the dCTR based on the prediction using MobiDB-lite 5.0 (https://mobidb.bio.unipd.it/). Besides, variations in sequences also exist in the L2, L3, and LC regions (Fig. 1B). *Tr*AA14A shared only 21.88% and 23.26% sequence identities with the full-length sequences of *Pc*AA14A and *Pc*AA14B leaving out signal peptides, and the corresponding values for *Tr*AA14B were 16.63% and 15.68%, respectively (Fig. 1B). The sequence identities of *Tr*AA14A with only catalytic domains of *Pc*AA14A and *Pc*AA14B after removal of 130-aa C-extension could reach up to 39.04% and 41.04%, and the corresponding values for *Tr*AA14B were 21.88% and 23.26%, respectively. The first N-terminal histidine together with second His (H116 and H119 for *Tr*AA14B and *Tr*AA14B, respectively) and Tyr (Y192) residues were conserved among all proteins, which form a copper-binding histidine brace coordination environment. Significantly, *Tr*AA14A has extra sequences (such as WSGTGTPPGCIQDD) between Asn82 and Ile83, but devoid of a sequence GADPDHGKP between Cys202 and Asp212 corresponding to *Pc*AA14B. A similar phenomenon was also found in *Tr*AA14B. Of note, *Tr*AA14A shared only 44.53% identity with *Tr*AA14B, and many variations in sequence also exist between them. These sequence variations reflected the diversity of AA14 LPMO-encoding sequences in different fungi or even in a single fungus.

Since few AA9 LPMOs, such as *Mt*LPMO9A from *Myceliophthora thermophila* [15], *Mc*LPMO9H from *Malbranchea cinnamomea* [23], and *Nc*LPMO9F and *Nc*LPMO9L from *Neurospora crassa* [24] also showed cellulose-associated xylan-activity, we conducted sequence alignment of AA14 LPMOs (*Pc*AA14A, *Pc*AA14B, *Tr*AA14A and *Tr*AA14B) with these previously characterized xylan-active AA9 LPMOs. It could be found that both *Tr*AA14A and *Pc*AA14B exhibit more extended L2 and LC regions but shorter LS than xylan-active AA9 LPMOs (Additional file 1: Fig. S1).

### Expression and purification of *Tr*AA14A

Both *Tr*AA14A- and *Tr*AA14B-encoding genes were synthesized and transformed into *P. pastoris*, but only *Tr*AA14A was successfully expressed and purified by Ni–NTA system. SDS-PAGE analysis revealed that the molecular weight of recombinant *Tr*AA14A was approximately 60 kDa, which was twice that of the deglycosylated form (approximately 30 kDa) after Endo H treatment (Additional file 1: Fig. S2A). NetNGlyc 1.0 analysis (http://www.cbs.dtu.dk/services/NetNGlyc/) indicated it contains three potential N-glycosylation sites, so the increase in molecular weight of recombinant *Tr*AA14A may be due to over-glycosylation usually occurred in the expression of LPMOs in *P. pastoris*. In addition, the purity and molecular weights of recombinant *Nc*LPMO9C and *Ep*LPMO9A from *Neurospora crassa* and *Eupenicillium parvum* 4–14, respectively, were also confirmed by SDS-PAGE (Additional file 1: Fig. S2B).

### Kinetic constant analysis and H_2_O_2_ production of *Tr*AA14A and apo-*Tr*AA14A

Kinetic constant analyses of *Tr*AA14A demonstrated that the *k*_*cat*_ for H_2_O_2_ was 2.95 s^−1^ and *K*_*m*_ was 0.26 mM, when the concentration of 2, 6-DMP was set to 1.0 mM (Additional file 1: Fig. S3A), while the *k*_*cat*_ for 2, 6-DMP was 1.32 s^−1^ and *K*_*m*_ was 0.79 mM, when the H_2_O_2_ concentration was set to 100 μM (Additional file 1: Fig. S3B). It should be worth noting that it is insufficient kinetics analysis because of lower concentration of H_2_O_2_ than* K*_*m*_ (0.26 mM) used in this study, more detail kinetics analysis needs to be carried out in the future. Under the same reaction condition, no activity of apo-*Tr*AA14A towards 2, 6-DMP was detected, indicating that removal of Cu^2+^ resulted in a complete loss of its peroxidase activity. H_2_O_2_ was produced when *Tr*AA14A was incubated with ascorbic acid (AscA) in the absence of substrates, and the concentration H_2_O_2_ rapidly accumulated to 2.9 µM during the initial 10 min, then slowly increased to 4.4 µM after 40 min of reaction. As expected, no H_2_O_2_ was produced by apo-*Tr*AA14A, further confirming that its activity was eliminated due to the removal of Cu^2+^ (Additional file 1: Fig. S3C).

### Oxidizing activity and synergism of *Tr*AA14A on cellulosic substrates

**Fig. 2:**
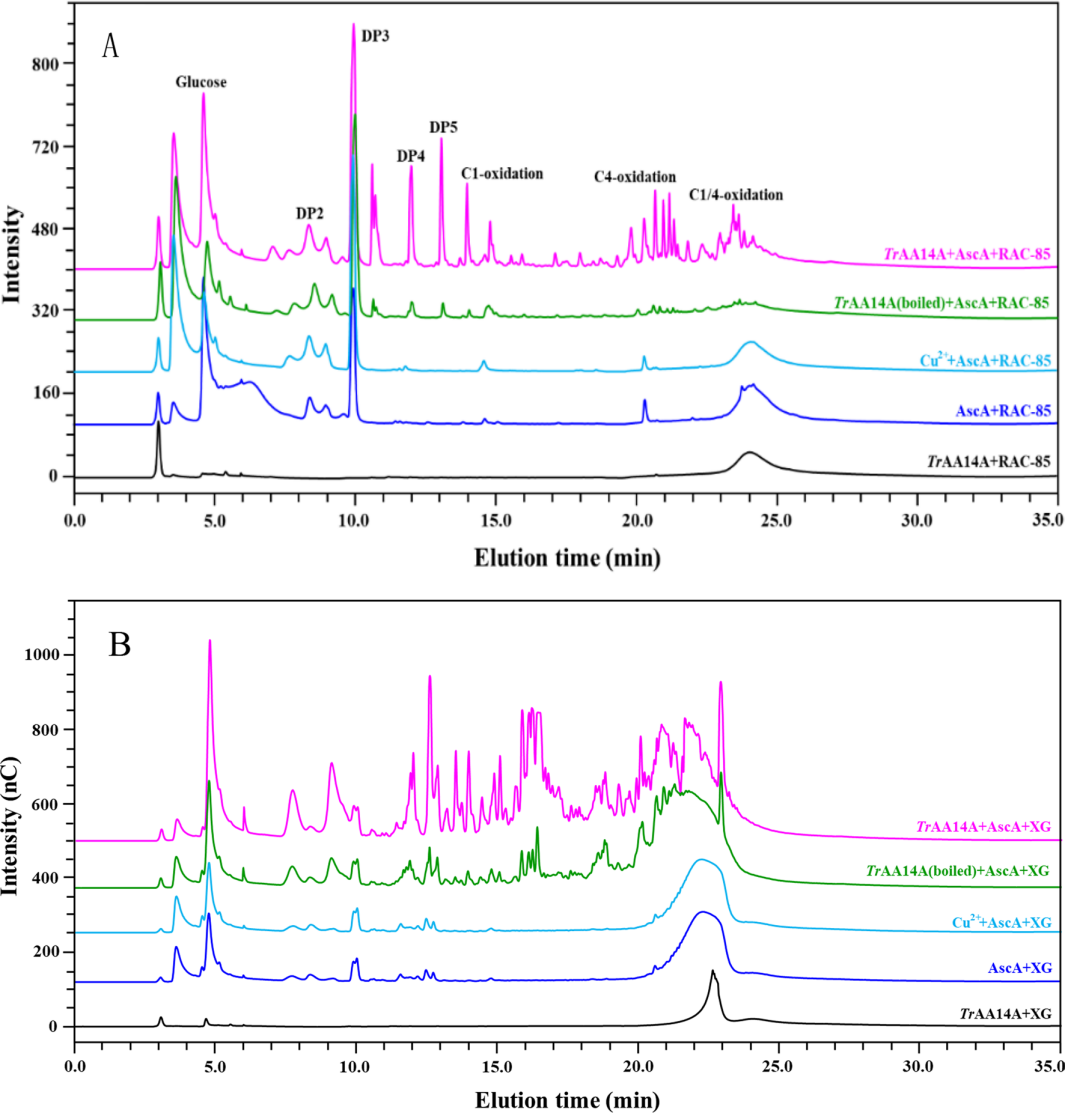
HPAEC-PAD analysis of reaction products generated by *Tr*AA14A from RAC-85 and xyloglucan. **A** The reaction products generated from RAC-85. **B** The reaction products generated from xyloglucan. The oxidative activities of *Tr*AA14A towards cellulosic substrates or xyloglucan were determined in the reaction mixture (2.0 mL) containing various substrates (5 mg), 1 μM *Tr*AA14A and 1 mM AscA in sodium acetate buffer (pH 5.0, 50 mM) in an incubator at 45 ℃ and 200 rpm for 24 h. The control reaction containing various substrates (5 mg) with AscA (1 mM), or AscA (1 mM) and Cu^2+^ (1 μM), or heat-inactivated *Tr*AA14A (designated *Tr*AA14A(boiled), boiled at 99 °C for 15 min) (1 μM) and AscA (1 mM) was also performed in parallel under the same condition

HPAEC-PAD (High performance anion exchange chromatography equipped with pulsed amperometric detection) analysis of reaction products clearly demonstrated that *Tr*AA14A with 1 mM AscA could generate nonoxidized, C1-(~ 12.5–18 min), C4-(~ 18–23 min) and C1/C4-oxidized (~ 23–25 min) cello-oligosaccharides from regenerated amorphous cellulose (RAC-85) [25, 26], while no nonoxidized or oxidized cello-oligosaccharides were detected from the control samples incubated RAC-85 with *Tr*AA14A, or AscA, or AscA and Cu^2+^ only (Fig. 2A). This result clearly demonstrated that *Tr*AA14A had oxidative activity towards RAC-85. Interestingly, the heat-inactivated *Tr*AA14A still showed weak oxidative activity towards RAC-85, although *Tr*AA14A was boiled at 99 °C for 15 min. Very weak oxidative activity towards Avicel was observed for *Tr*AA14A (Additional file 1: Fig. S4A). *Tr*AA14A also showed tiny oxidative activity towards mercerized fiber (Additional file 1: Fig. S4B), however, it did not have oxidative activity on α-cellulose (Additional file 1: Fig. S4C). The cellulose-activity of *Tr*AA14A was also compared with other characterized AA9 LPMOs under the same condition. Based on the peak areas of native sugars and C4-oxidized products, it was estimated that the oxidative activity of *Tr*AA14A towards RAC-85 was approximately 4.8-fold and 1.4-fold lower than *Nc*LPMO9C and *Ep*LPMO9A, respectively (Additional file 1: Fig. S5). The residual oxidative activity on RAC-85 was also observed for heat-inactivated *Nc*LPMO9C even though it was boiled at 99 °C for 30 min. In contrast, no residual oxidative activity was retained for heat-inactivated *Ep*LPMO9A after boiled at 99 °C for 15 min (Additional file 1: Fig. S5). Notably, we found that the heat-inactivated *Nc*LPMO9C and *Tr*AA14A were still soluble while heat-inactivated *Ep*LPMO9A was precipitated after boiled at 99 °C for 15 min, so no soluble protein in the supernatant was detected by SDS-PAGE analysis (Additional file 1: Fig. S2B). In comparison, *Tr*AA14A had similar oxidative activity towards Avicel as *Ep*LPMO9A, however, its oxidative activity towards Avicel was significantly lower (threefold) than *Nc*LPMO9C (Additional file 1: Fig. S5).

**Fig. 3:**
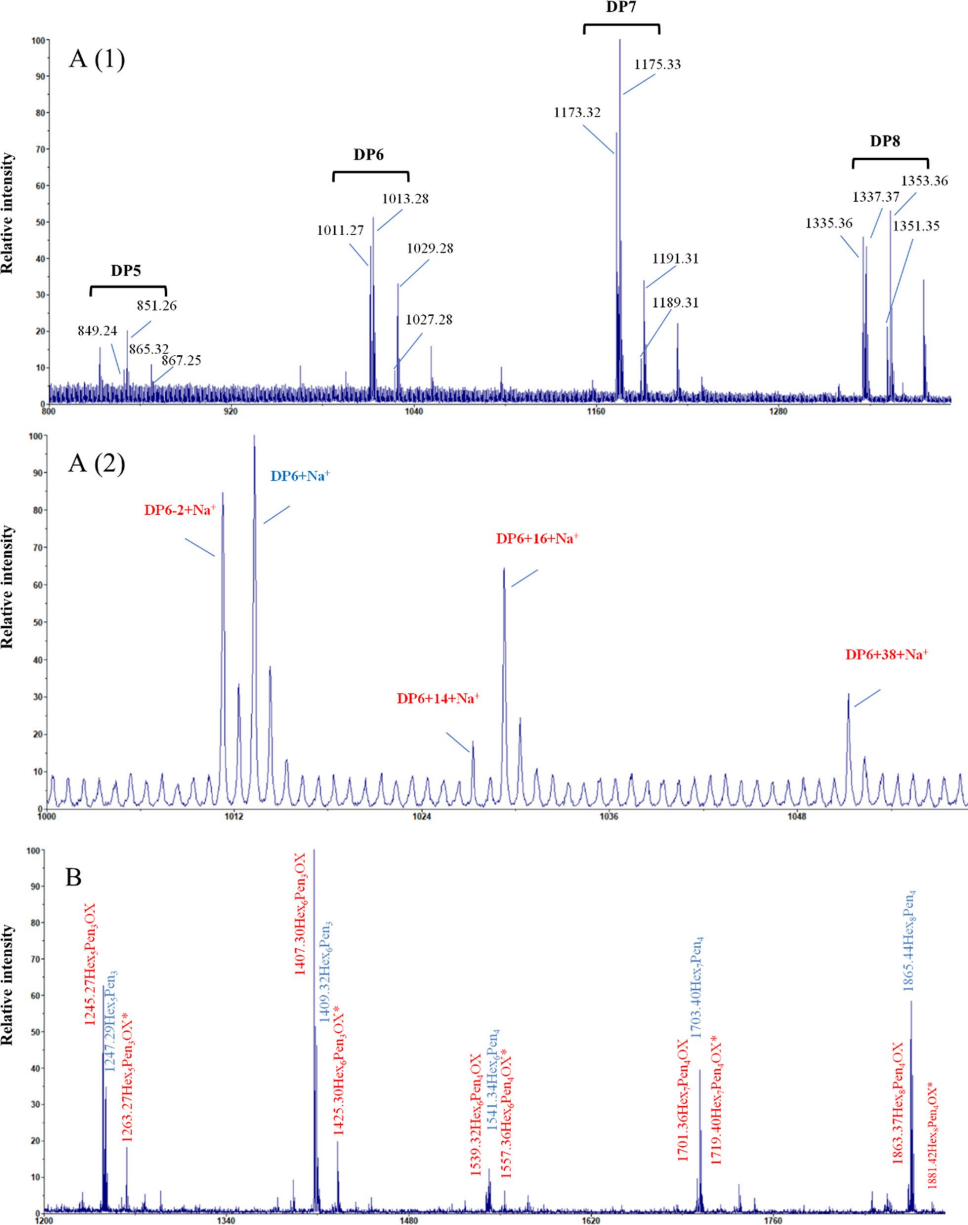
MALDI-TOF MS analysis of the products from RAC-85 (**A**) and xyloglucan (**B**) by *Tr*AA14A. nonoxidized and oxidized products are shown in blue and red letters, respectively. Hex, hexose; Pen, pentose; OX, C4 oxidized product; OX*, C1-oxidized product in the aldonic acid form or C4-oxidized product in the gemdiol form. The oxidative reaction of *Tr*AA14A towards RAC-85 and xyloglucan was performed in the reaction mixture (2.0 mL) containing various substrates (5 mg), 1 μM *Tr*AA14A and 1 mM AscA in sodium acetate buffer (pH 5.0, 50 mM) in an incubator at 45 ℃ and 200 rpm for 24 h

To identify the nonoxidized and oxidized sugars released from RAC-85 by the *Tr*AA14A, the reaction products were analyzed by matrix-assisted laser desorption/ionization-time-of-fight mass spectrometry (MALDI-TOF MS) (Fig. 3). The monocharged sodiated molecules of the nonoxidized cello-oligosaccharides with a degree of polymerization ranging from DP5 to DP8 appear as a homologous series of peaks at *m*/*z* 851, 1013, 1175, and 1337. The monocharged sodiated molecules of C1-oxidized cello-oligosaccharides in the lactone form or C4-oxidized cello-oligosaccharide in the keto-aldose form ranging from DP5 to DP8 appear as a homologous series of peaks at *m*/*z* 849, 1011, 1173, and 1335. However, they are generally annotated as the latter, since the aldonic acid–lactone equilibrium of C1-oxidized products is strongly shifted towards aldonic acid in MALDI-TOF MS, yielding weak lactone signals [27]. The monocharged sodiated molecules of C1-oxidized cello-oligosaccharides in the aldonic acid form or C4-oxidized cello-oligosaccharide in the gemdiol form ranging from DP5 to DP8 appear as a homologous series of peaks at *m*/*z* 867, 1029, 1191, and 1353. The monocharged sodiated molecules of doubly oxidized cello-oligosaccharide in the keto-aldose and aldonic acid form ranging from DP5 to DP8 appear as a homologous series of peaks at *m*/*z* 865, 1027, 1189, and 1351 [Fig. 3A(1)]. A distinct signal with *m*/*z* 1051 is the characteristic peak of sodium salt of the sodiated molecules of C1-oxidized cellohexaose in the aldonic acid form [DP6 + 38 + Na^+^] [Fig. 3A(2)]. On basis of HPAEC-PAD chromatography pattern and MALDI-TOF MS spectra analyses, it could be concluded that *Tr*AA14A has oxidative cleavage activity on cellulose at C1-carbon, and probably also at C4-carbon. However, more sophisticated experiments with MALDI-TOF MS or even other method should be used to determine the definite regioselectivity of *Tr*AA14A in the future [27, 28].

**Fig. 4:**
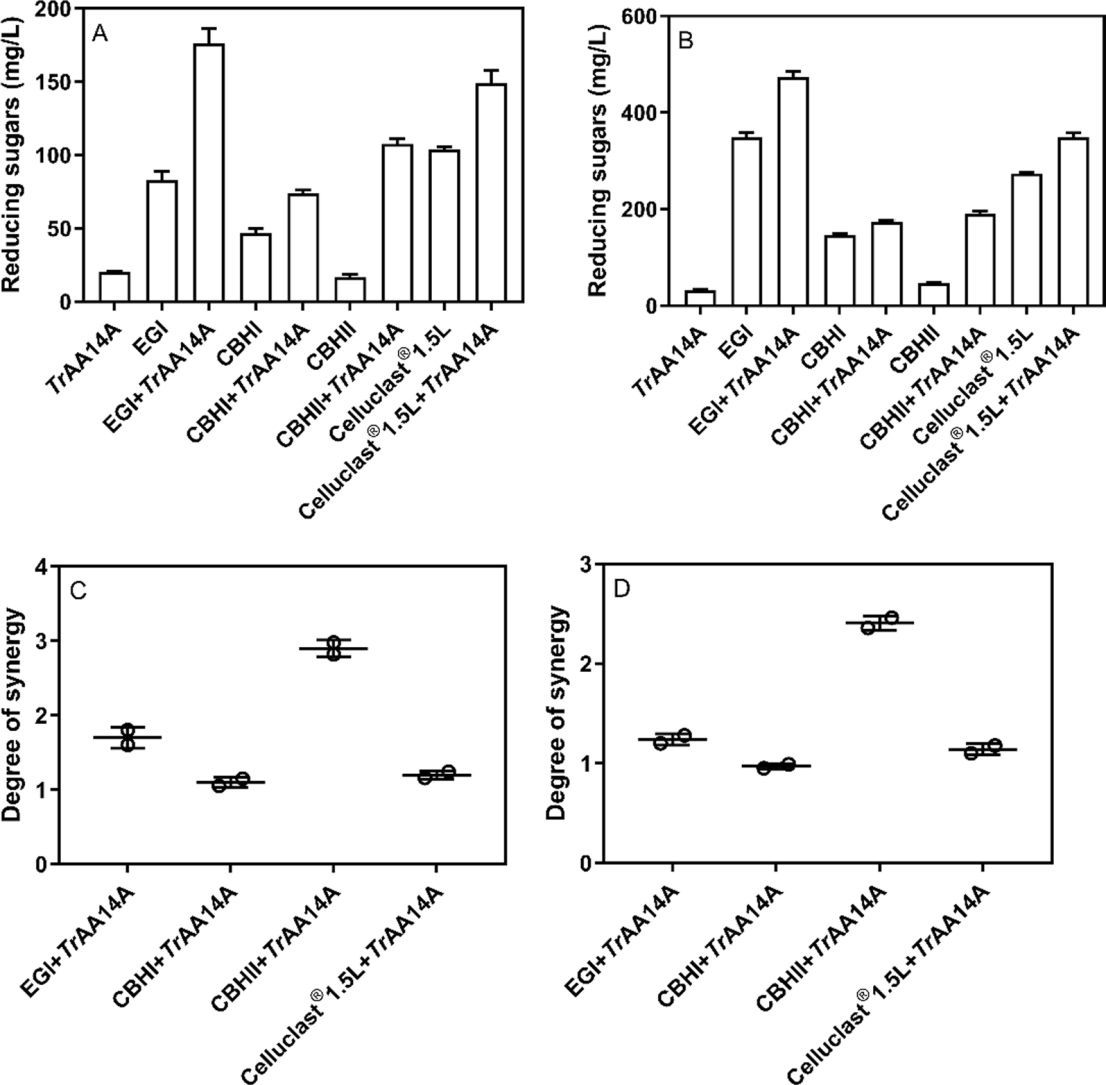
The synergy between *Tr*AA14A and GHs on cellulosic substrates. **A** The released reducing sugar from the synergy between *Tr*AA14A and GHs on mercerized fiber. **B** The released reducing sugar from the synergy between *Tr*AA14A and GHs on RAC-85. **C** The degree of synergy (DS) for *Tr*AA14A with GHs on mercerized fiber. **D** The degree of synergy (DS) for *Tr*AA14A with GHs on RAC-85. The synergism of *Tr*AA14A with GHs was performed in a reaction mixture (1 mL) by mixed incubation of *Tr*AA14A (1 μM) with EGI (10 μg), CBHI (20 μg), CBHII (20 μg), or Celluclast®1.5L (0.04 U), and 4 mg/mL RAC-85 or mercerized fiber, with 1 mM AscA in sodium acetate buffer (pH 5.0, 50 mM) at 45 ℃ and 1000 rpm for 1 h in a thermomixer. The control reaction in a reaction mixture (1 mL) containing individual *Tr*AA14A (1 μM), EGI (10 μg), CBHI (20 μg), CBHII (20 μg), or Celluclast®1.5L (0.04 U), and 4 mg/mL RAC-85 or mercerized fiber, with 1 mM AscA was performed under same condition

Since *Tr*AA14A displayed a strong oxidative activity towards cellulosic substrates similar as *Ep*LPMO9A, we further explored the synergistic action of *Tr*AA14A with GHs on the hydrolysis of RAC-85 and mercerized fiber, respectively (Fig. 4A and B). As shown in Fig. 4, the synergy degrees could reach up to 1.70, 1.10, 2.90 and 1.20, or 1.24, 0.97, 2.41 and 1.14, when *Tr*AA14A synergistically acted with EGI, CBHI, CBHII and Celluclast®1.5 L on mercerized fiber, or RAC-85, respectively (Fig. 4C and D). Overall, *Tr*AA14A showed the highest synergistic action with CBHII, followed by EGI and Celluclast®1.5 L. In contrast, *Tr*AA14A exhibited relatively weaker or even no synergistic effect with CBHI.

### Oxidative activities of *Tr*AA14A on hemi/cellulosic substrates

**Fig. 5:**
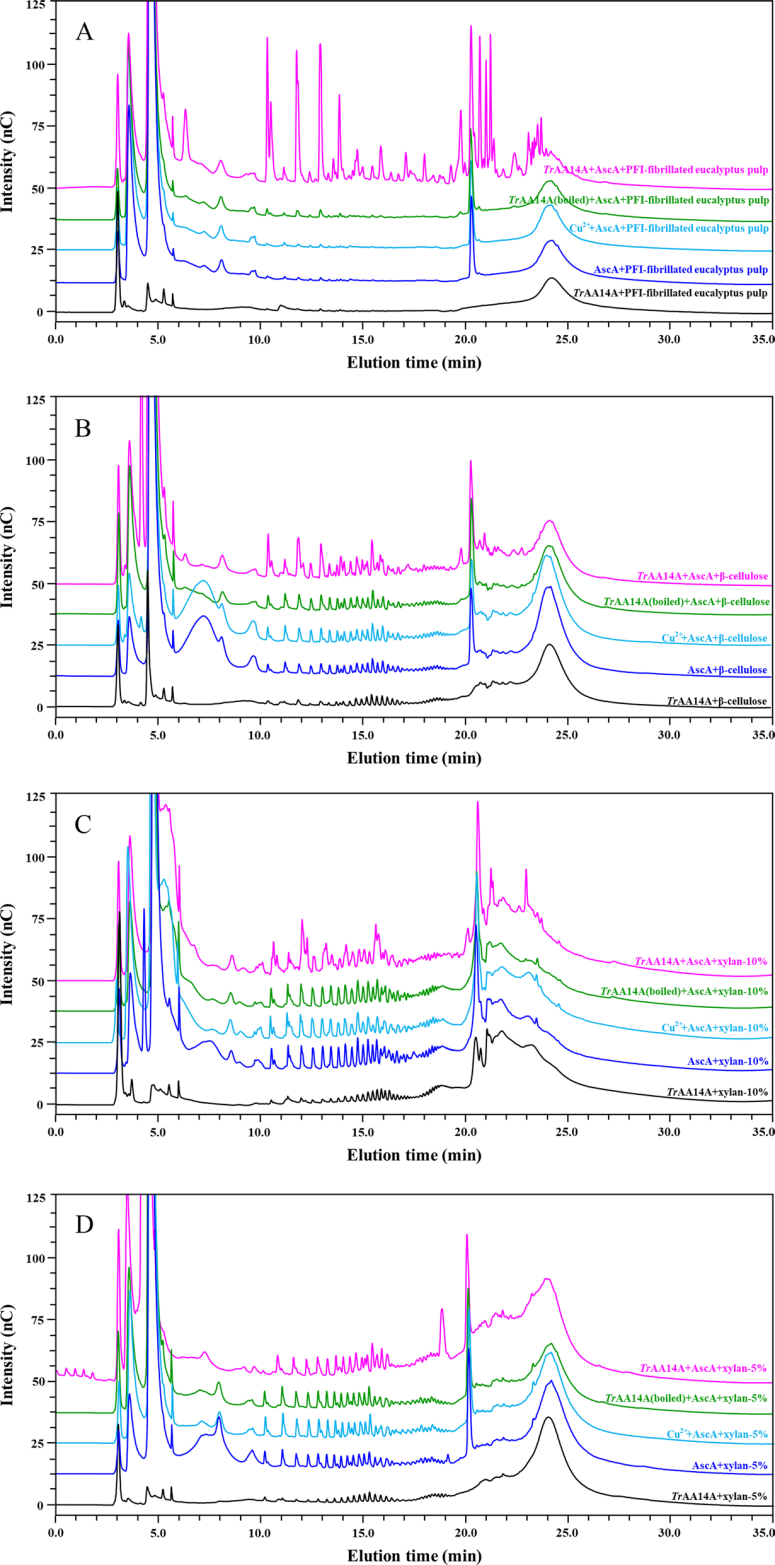
HPAEC-PAD analysis of reaction products generated by *Tr*AA14A from different hemi/cellulosic substrates. **A** PFI-fibrillated eucalyptus pulp. **B** β-Cellulose. **C** Xylan-10%. **D** Xylan-5%. The reaction was performed in the reaction mixture (500 μL) containing 5 mg substrate, *Tr*AA14A (1 μM) and AscA (1 mM) in sodium acetate buffer (pH 5.0, 50 mM) in a thermomixer at 45 ℃ and 1000 rpm for 1 h. The control reaction containing various substrates (5 mg) with AscA (1 mM), or AscA (1 mM) and Cu^2+^ (1 μM), or heat-inactivated *Tr*AA14A [designated *Tr*AA14A(boiled), boiled at 99 °C for 15 min] (1 μM) and AscA (1 mM) was also performed in parallel under the same condition. After reaction, the reaction solution was immediately filtered by passing the reaction mixture through a membrane with a pore size of 0.22 μm and analyzed by HPAEC-PAD

Various hemi/cellulosic substrates were used to assess the substrate specificities of *Tr*AA14A. Besides cellulosic substrates, HPAEC analyses of reaction products revealed that *Tr*AA14A also showed strong oxidative activity towards xyloglucan (Fig. 2C), Analysis of HPAEC chromatograms revealed that some peaks were generated in the control samples replacing *Tr*AA14A with AscA or Cu^2+^ and AscA likely due to some side-reaction. However, the intensities and profiles of chromatograms for PFI-fibrillated eucalyptus pulp in the presence of reductant AscA were significantly stronger and different compared to the control reactions (Fig. 5A). Thus, it could be convincible that *Tr*AA14A showed oxidative activity towards PFI-fibrillated eucalyptus pulp. The difference in intensities and profiles of chromatograms particularly in the elution time of 15–22 min representatives of oxidation products was also observed for β-cellulose and xylan-10% by *Tr*AA14A compared to the control reactions (Fig. 5B and C), despite the difference became less significant than that on PFI-fibrillated eucalyptus pulp, indicating that *Tr*AA14A also had oxidative activity on these alkaline-extracted hemi/cellulosic substrates. However, it became less obvious for xylan-5% (Fig. 5D), and even no difference in HPAEC-PAD chromatogram patterns was observed between the5 reaction sample with the control reactions on commercial wheat arabinoxylan (WAX) (Additional file 1: Fig. S4D). The contents of neutral sugars and uronic acids of polysaccharides in these hemi/cellulosic substrates (except for xyloglucan) are listed in Additional file 1: Table S1. PFI-fibrillated eucalyptus pulp contains 67.46% glucose and 21.57% xylose arising mainly from cellulose and xylan, and also from xyloglucan in minor amount, respectively. α-Cellulose is the pulp fraction resistant to 17.5% and 9.45% NaOH solution pretreatment, so most of hemicellulose was extracted and only a very small amount of xylan was retained. In this study, the xylan-related samples including β-cellulose, xylan-10% and xylan-5% were obtained from PFI-fibrillated eucalyptus pulp by alkaline-extraction using different concentrations of NaOH with different times and then precipitated in acid conditions. It has been suggested that eucalyptus pulp contains two types of xylans differentiated in chemical structure, molecular weight and allocation [29]. The xylan located in the interfibrillar space, essentially in the outer layers of the cell wall could be extracted by 5% NaOH solution. Another xylan located in inner areas of the pulp fibers and laterally interacted with fibrils could be extracted only by higher NaOH concentration solution (~ 10%) because it needs higher swelling in alkaline solution to separate the fibrils. In addition to the solubilization of xylan, the low-molecular-weight cellulose chains from the amorphous and crystallite surface of fibrils on PFI-fibrillated eucalyptus pulp (even some extent of decomposed cellulose) were also co-solubilized into solution during extraction process but the extent was largely depended on the NaOH concentration and treatment time [29, 30]. So, besides xylan, varying contents of cellulose also exist in these samples. Glucose contents in β-cellulose, xylan-10% and xylan-5% are 44.40%, 44.13% and 1.39%, while xylose contents are 47.50%, 59.55% and 84.00%, respectively. In comparison, xylan-5% mainly contains xylan and very low fraction of cellulose, while β-cellulose and xylan-10% contain much higher contents of cellulose in addition to high fraction of xylan. It should be worth noting that the times of alkaline-extraction (with approximately 10% of NaOH solution) for xylan-10% is much longer than that for β-cellulose (2 h vs 0.5 h), Moreover, β-cellulose and xylan-10% were precipitated at pH 1.5 and pH 4.2, respectively, which may result in different coagulation and precipitation pattern of soluble xylan and cellulose fraction. This may be the reason for some differences of xylose and glucose contents between two samples. Besides, they also contain different contents of 4-O-methyl glucuronic acid (MeGlcA) and glucuronic acid (GlcA). This means that not only the extent of the xylan and cellulose or its molecular weight and purity, but also the structural heterogeneity of xylan or its network with cellulose may vary among different hemi/cellulosic substrates used in this study. These specific cellulose-heteroxylan network structures may exist more in PFI-fibrillated eucalyptus pulp, followed by β-cellulose and xylan-10%, whereas, such cellulose-heteroxylan network structure is much less in xylan-5% and even absent in WAX. This might explain why *Tr*AA14A has significantly higher oxidative activity towards PFI-fibrillated eucalyptus pulp than β-cellulose, xylan-10% and xylan-5%. In summary, the oxidative activity of *Tr*AA14A on hemi/cellulosic substrates declined as the content of xylan increased and no oxidative activity was observed on pure WAX, indicating that *Tr*AA14A preferred to decompose some specific network structures formed between cellulose and hemicellulose of the plant cell wall.

**Fig. 6:**
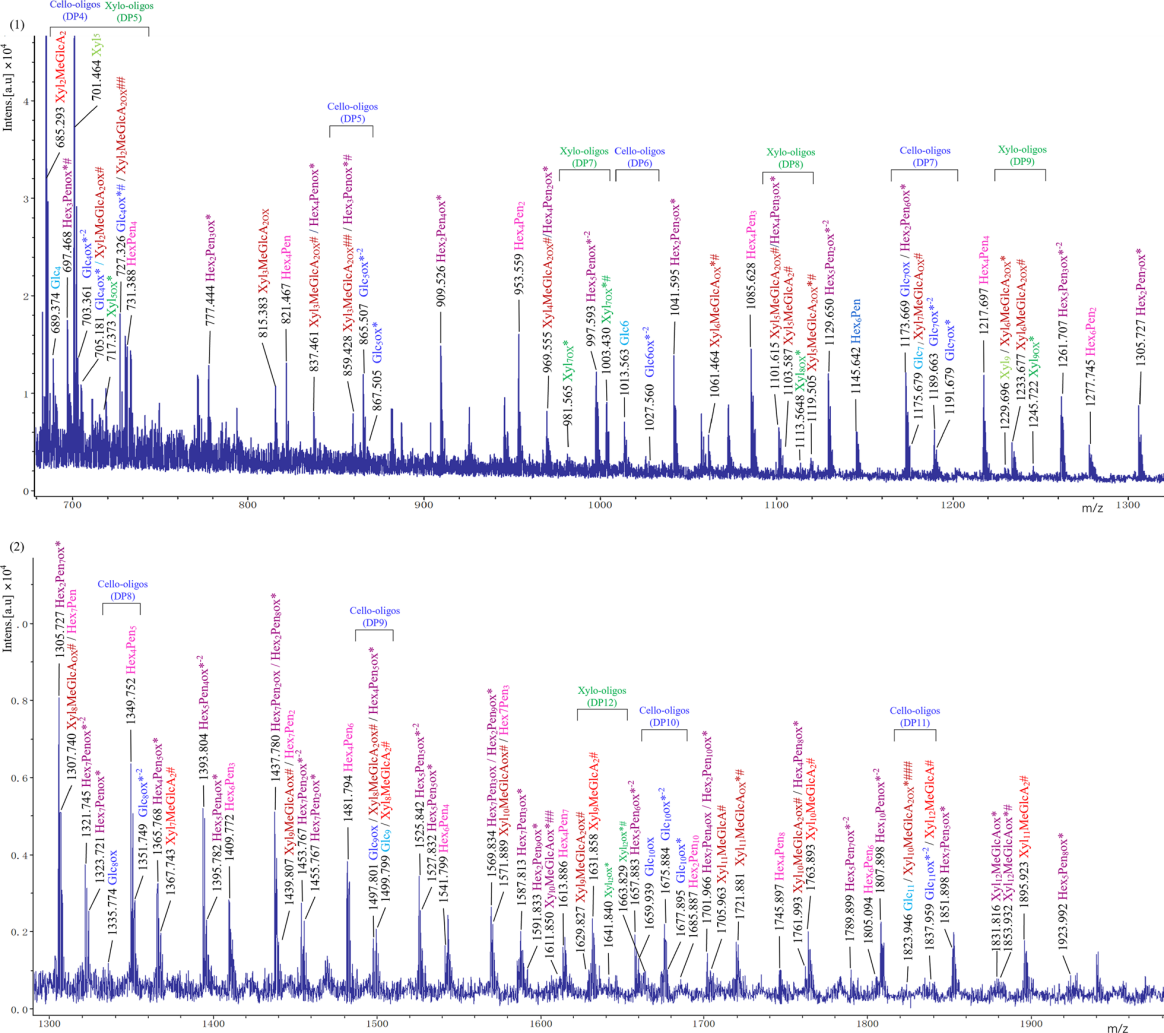
MALDI-TOF analysis of the products generated by *Tr*AA14A from PFI-fibrillated eucalyptus pulp. Blue: Cello-oligos; Dark blue: Oxidized cello-oligos. Light green: Xylo-oligos; Green: Oxidized xylo-oligos. Red: Xylo-oligos with methyl glucuronic acid; Dark red: Oxidized xylo-oligos with methyl glucuronic acid. Purple: XG-oligos; Dark purple: Oxidized XG-oligos. All labelled peaks indicate monocharged sodiated molecules. OX: C4 oxidized products; OX*^−2^: C1 and C4 double oxidation products. OX*: C1-oxidized product in the aldonic acid form or C4-oxidized product in the gemdiol form. #: Sodium salts of aldonic or alduronic acids. The reaction was performed in the reaction mixture (500 μL) containing 5 mg substrate, *Tr*AA14A (1 μM) and AscA (1 mM) in sodium acetate buffer (pH 5.0, 50 mM) in a thermomixer at 45 ℃ and 1000 rpm for 1 h

No obvious differences in HPAEC-PAD chromatogram patterns were observed from the reaction samples with substrate only or incubating substrate and *Tr*AA14A with or without AscA, indicating that *Tr*AA14A also had no oxidative activity or no soluble products could be generated from other commercial hemicellulosic substrates including glucomannan, lichenin (β-1,3–1,4-glucan) and beechwood xylan (BeWX) or beechwood xylan (BeWX)-RAC-85 mixture used in these experiments (Additional file 1: Fig. S6). Since *Tr*AA14A showed strong oxidative activity towards xyloglucan and PFI-fibrillated eucalyptus pulp based on HPAEC analyses, we further analyzed the profiles of nonoxidized and oxidized products by MALDI-TOF MS. For xyloglucan, the mass spectrum shows a wide range of xyloglucan oligosaccharides with masses corresponding to a series of sodium adducts of nonoxidized [e.g., Hex_5_Pen_3_ (*m*/*z* 1247)], C4-oxidized products (4-ketoaldose form) [e.g., Hex_5_Pen_3_OX (*m*/*z* 1245)], C1-oxidized (aldonic acid form) or C4-oxidized (gem-diol form) [e.g., Hex_5_Pen_3_OX* (*m*/*z* 1263)], respectively) (Fig. 3B). The product profiles of *Tr*AA14A were similar to *At*AA9B from *Aspergillus tamarii* [31], indicating that *Tr*AA14A could oxidatively cleave the glucan backbone regardless of side substitutions. Both series of nonoxidized and oxidized cello-oligosaccharides and xylo-oligosaccharides (nonsubstituted or substituted with methyl glucuronic acid) were detected from PFI-fibrillated eucalyptus pulp (Fig. 6), indicating that *Tr*AA14A is able to simultaneously oxidize cellulose and xylan in natural hemi/cellulosic substrate. It is noteworthy that putatively nonoxidized and oxidized Hex_n_Pen_m_ were detected from PFI-fibrillated eucalyptus pulp. This result suggested that xyloglucan may co-exist with cellulose and xylan in this substrate, and can be efficiently cleaved by oxidative action of *Tr*AA14A.

### Structure modelling of *Tr*AA14A

**Fig. 7:**
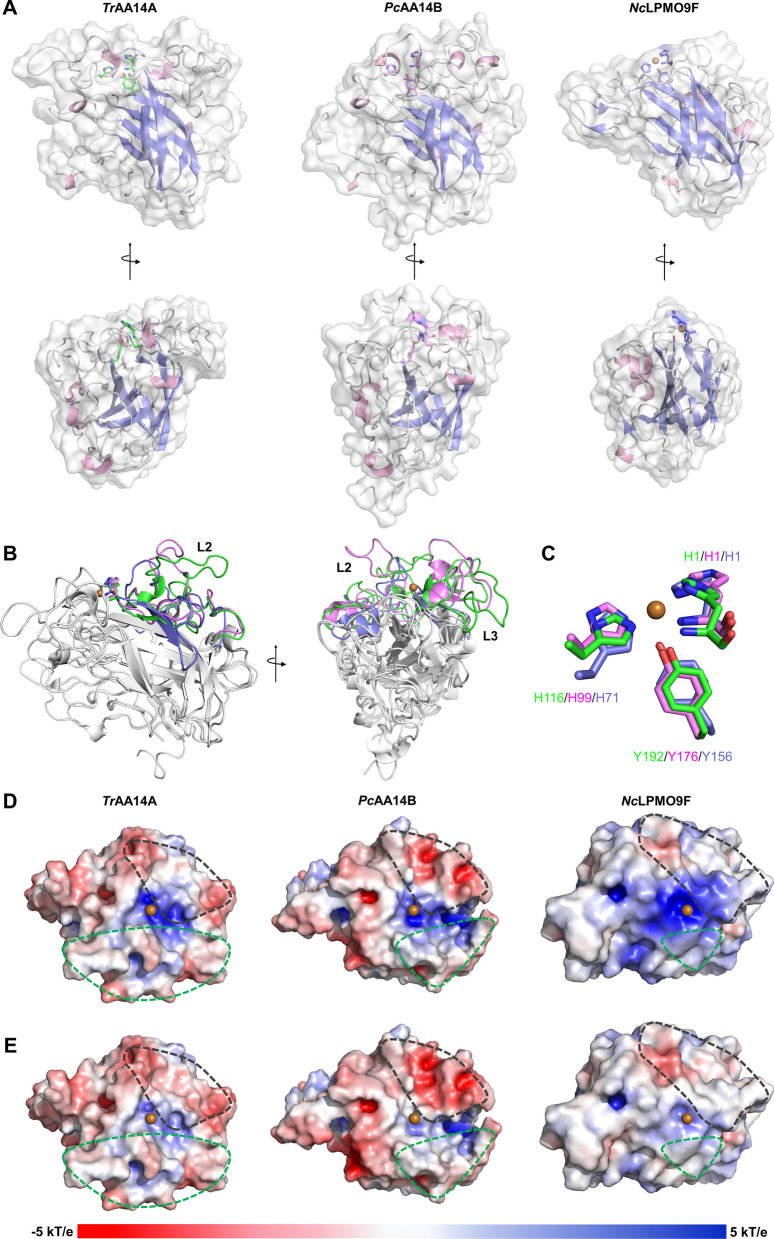
Comparison of structure and electrostatic potential of *Tr*AA14A predicted by Alphafold 2 with *Pc*AA14B (PDB ID: 5NO7) and the previously characterized xylan-active *Nc*LPMO9F (PDB ID: 4QI8). **A** The three-dimensional structure of *Tr*AA14A *Pc*AA14B and *Nc*LPMO9F in two views related by a 90° rotation to visualize of the loop, helix and sheet decorated by white, pink and slate carton, respectively. **B** Comparison of *Tr*AA14A (gray and green), *Pc*AA14B (gray and violet) and *Nc*LPMO9F (gray and slate), and displayed in two views related by a 90° rotation. **C** Active site residues overlay of *Tr*AA14A (green), *Pc*AA14B (violet), and *Nc*LPMO9F (slate). **D**, **E** The electrostatic potential calculated for *Tr*AA14A, *Pc*AA14B and *Nc*LPMO9F at pH 5.0, and pH 6.0, respectively. The electrostatic potential is shown as color gradient from red to blue (− 5 to + 5 kT/e), and the catalytic center Cu is shown as brown sphere. The gray circle indicates the L2, and the green circle indicates the L3

The 3D structural model of *Tr*AA14A was constructed by submitting the amino acid sequence to the AlphaFold2, and SWISS-MODEL web servers, respectively, and model quality was evaluated via QMEAN. The result from AlphaFold2 was ultimately selected as it had higher scores for QMEAN4 and QMEANDisco, with values of 0.3 and 0.67 ± 0.05, respectively, in comparison, which were higher than the − 3.43 and 0.65 ± 0.05 of the SWISS-MODEL simulated structure. (The score of a higher value signifies a model with a higher confidence and vice-versa). The core of *Tr*AA14A predicted by AlphaFold2 consists of an anti-parallel immunoglobulin G-like β-sandwich structure and a canonical His-brace motif amongst all LPMOs families (Fig. 7A).

*Tr*AA14A exhibits a relatively flat active-site surface, which is distinct from *Pc*AA14B with a clamp fashioned through two distinguished surface loops equivalent to L2 and L3 regions of AA9 LPMOs, but more similar to the flat active-site surfaces in AA9 LPMOs such as *Nc*LPMO9F (PDB ID: 4QI8). The structural comparison revealed that the differences in active-site surface mainly lie in the L2 region of *Tr*AA14A (residues 12–89), *Pc*AA14B (residues 12–74) and *Nc*LPMO9F (residues 10–53) (Fig. 7B). The L2 region forms a loop segment in *Pc*AA14B, in contrast, the N-terminal part of *Tr*AA14A of L2 region makes up a short β-strand segment (single β-strand or a β-hairpin) similar as that in *Nc*LPMO9F. In addition, *Tr*AA14A contains a short α-helix insertion in the L2 region, whereas no corresponding helix was found in the structures of *Pc*AA14B and *Nc*LPMO9F. The predicted active site of *Tr*AA14A is constituted with two histidine (His1 and His116) and a tyrosine (Tyr192), forming the canonical histidine brace (Fig. 7C). The His-brace of *Tr*AA14A was superimposed well with *Pc*AA14B except for a slight directional deviation in the His 1 position (Fig. 7C), indicating that the copper-binding histidine brace was conserved among LPMOs belonging to different AA families. The surface electrostatic potentials of *Tr*AA14A, *Pc*AA14B and *Nc*LPMO9F were also compared at pH 5.0 and pH 6.0, which are commonly used for assaying their oxidative activity. Despite positive charges are heavily concentrated nearby the region closely around Cu^2+^ at pH 5.0 in three enzymes, the electrostatic potentials on L2 and L3 surface are remarkably different (Fig. 7D). The predominate potentials on L2 surface of *Pc*AA14B and *Nc*LPMO9F are negative and positive, respectively, while L2 surface of *Tr*AA14A is largely neutral with slight negatively charged patches (Fig. 7D). The entire L3 surfaces of *Pc*AA14B and *Nc*LPMO9F tend to be weakly positive, while the L3 surface of the *Tr*AA14A is distributed like patches with slight positive and negative signs. At pH 6.0, the neutralization of positive charge on protein surface especially around Cu^2+^due to de-protonation change their states to more negatively charged than that at pH 5.0. It was regarded that L2 together with L3, LS and LC regions contribute to shaping the substrate-binding surface. So, we supposed that the differences in substrate specificities among *Tr*AA14A, *Pc*AA14B and *Nc*LPMO9F may be attributed to the variations in substrate-binding surfaces [7].

## Discussion

In the present study, we firstly identified a novel enzyme *Tr*AA14A from ascomycete *T. rugulosus*, which has the unique ability to simultaneously oxidize cellulose, xylan and xyloglucan in natural hemi/cellulosic substrate at C1-carbon, and probably also at C4-carbon. *Tr*AA14A also exhibited a synergistic action with GHs on cellulosic substrates. So far, two previously characterized AA14 LPMOs *Pc*AA14A and *Pc*AA14B did not show any cellulose- or xylan-oxidizing activity, but only C1-oxidized xylotriose (X3ox) and xylotetraose (X4ox) and nonoxidized xylooligosaccharides substituted with methyl glucuronic acid (X3MeGlcA, X4MeGlcA and X5MeGlcA) could be detected when synergistically acting with GH11 xylanase [16]. So, these unusual properties of *Tr*AA14A are distinguished from previously characterized AA14 LPMOs from basidiomycete *P. coccineus*, despite *Tr*AA14A is strongly classified as AA14 LPMOs according to phylogenetic analysis and showing approximately 40% identity with *Pc*AA14B. On the other hand, the C1 or C4-oxidizing cellulose and xyloglucan activity, and boosting effect on CBHII and EGI activity made *Tr*AA14A very similar to the C1/C4-oxidizing AA9 LPMOs such as *Ao*LPMO9A and *Ao*LPMO9B from *Aspergillus oryzae*, because both of C1/C4-oxidizing AA9 LPMOs boosted CBHII and EGI activity [25]. The synergism with CBHII was also reported for other C1/C4-oxidizing AA9 LPMOs such as *Ta*AA9A and *Ls*AA9A from *Thermoascus aurantiacus* and *Lentinus similis*, respectively [32]. Cellulose is not in an isolated form but in an aggregated form in an aqueous buffer, and different cellulose substrates (such as α-cellulose, Avicel, mercerized fiber, and RAC) have different aggregation structures with different crystallinities. Of note, despite *Tr*AA14A had strong oxidative activity on RAC-85 similar as AA9 LPMO, it showed weak oxidative activity towards Avicel or even did not degrade the other type of cellulose like α-cellulose, indicating that a limited type of celluloses with less crystallinity or specific aggregation structures (such as RAC) can be the substrate of *Tr*AA14A.

Usually, AA9 LPMOs display strict oxidative activity on cellulose, only a few of their members (mainly C-oxidizing) have xylan-activity only when the xylan is combined with phosphoric acid-swollen cellulose (PASC) [15, 23, 24]. Although In-depth-studies suggested that AA9 LPMOs with xylan-activity on cellulose-bound xylans may be more widespread than previously imagined in the vast arsenal of AA9 family in response to the complex structures in plant cell walls [10, 33], to the best of our knowledge, LPMOs with extensive cellulose- and xylan-activity on natural hemi/cellulosic substrate had not been reported previously. For example, C1-oxidizing *Tt*LPMO9A and *Tt*LPMO9E have xylan-activity on PASC-beechwood xylan (BeWX) and PASC-birch acetylated glucuronoxylan mixtures. However, when sulfite pulped spruce fiber was used as a natural cellulosic substrate, only very weak signals corresponding to oxidized xylan-derived products could be detected in HPAEC-PAD chromatograms, indicating that their key functional role may be exclusively related to cellulose biodegradation and modification [33]. Of note, besides the phylogenetical diversity between AA9 and AA14 LPMOs, these two-family enzymes also shared difference in modularity. A fraction of AA9 LPMOs is modular proteins with a catalytic domain (CD) and a carbohydrate-binding module 1 (CBM1) connected together by a flexible linker peptide, while none of the AA14 members identified in fungal genomes harbors a CBM1 [16]. The presence of CBM1 was considered as the important indicator of Carbohydrate-Active enZYmes which may be directly involved in the degradation of crystalline cellulose or some recalcitrant polysaccharides associated with cellulose microfibrils. In view of the difference in domain architecture between AA9 LPMOs and AA14 LPMOs, it is reasonable to speculate that AA14 LPMOs may not target the recalcitrant xylan bound to cellulose microfibrils, instead, they may involve in catalysis of the cleavage of some type of hemicellulose-cellulose matrix in lignocellulose. So, in this study, we selected and used various hemi/cellulosic substrates with different contents of cellulose and xylan for assaying the oxidative activity of AA14 LPMOs. *Tr*AA14A could simultaneously oxidize cellulose, xylan and xyloglucan in natural hemi/cellulosic substrate such as PFI-fibrillated eucalyptus pulp, while the cellulolytic/hemicellulolytic activity became less significant on β-cellulose and xylan-10%, and even disappeared on WAX. The hemicellulose-cellulose network structure in natural hemi/cellulosic substrate such as PFI-fibrillated eucalyptus pulp might be different from that in the artificial xylan-cellulose mixture (such as birchwood xylan- or beechwood xylan-cellulose mixture) commonly used for assaying the xylan-activity of AA9 LPMOs [23, 24, 33], because *Tr*AA14A also did not display xylan-activity on BeWX-RAC-85 mixture. So, it is conceivable that *Tr*AA14A is distinctively different from the previously characterized xylan-active AA9 LPMOs and may have functionality in decomposing some specific hemicellulose-cellulose network structures in plant cell walls. However, in this study, we also found that *Tr*AA14A did not show xylanolytic activity towards commercial pure xylans such as WAX and BeWX, indicated that the xylan-oxidizing activity of *Tr*AA14A might also require the co-existence of cellulose to some extent. In-depth investigation using various sources of natural hemi/cellulosic substrates needs to be carried out in the future to elucidate the substrate profiles of *Tr*AA14A.

The current findings raise several questions what are the molecular determinants of substrate specificity and the functionality of AA14 LPMOs from ascomycete fungi. The molecular basis for LPMO substrate specificity is still poorly understood to date. It is considered that substrate specificity of LPMOs depends on multiple residues located on and near the substrate-binding surface [34, 35]. L2 loop is the most diverse region differing in length as well as secondary structure in various LPMOs, together with L3, LS and LC regions, shaping the substrate binding surface [7, 36, 37]. Alignment of AA14 LPMOs with AA9 LPMOs revealed that both *Tr*AA14A and *Pc*AA14B exhibit longer L2 and LC-equivalent regions than xylan-active AA9 LPMOs. Of note, by comparing the predicted structural model of *Tr*AA14A with AA9 and AA14 LPMOs, we found that the topography of the substrate-binding surface of *Tr*AA14A resembles AA9 LPMOs more closely than AA14 LPMOs. However, the electrostatic potential on L2 and L3 surfaces is more different from xylan-active *Nc*LPMO9F, and relatively closer to *Pc*AA14B. These findings indicate that *Tr*AA14A is distinctively from previously characterized xylan-active AA9 and AA14 LPMOs, and may explain why *Tr*AA14A has extensive cellulolytic/hemicellulolytic activity. In nature, the recalcitrance of the plant cell wall primarily arises from the complex and heterogeneous structure in which cellulose microfibrils are embedded in a matrix of hemicellulose and lignin, with crosslinking between the polymers [37–40]. Most components of fungal cellulolytic enzyme machinery have single substrate specificity not only for glycoside hydrolases but also for LMPOs. Therefore, the biodegradation of plant cell walls demands a large set of enzymes with varied substrate specificities that work in synergy to decompose three major polymeric components including cellulose, hemicelluloses, and lignin. It should be noteworthy that most of lignocellulosic biomass-degrading fungi usually have multiple AA9 LPMO-encoding genes in their genomes, while the number of AA14 LPMO-encoding genes is much lower. For example, *P. coccineus* has sixteen AA9 LPMO-encoding genes but only four AA14 LPMO-encoding genes [16]. It is widely regarded that the multiplicity of AA9 LPMOs in a single fungus may provide a better advantage to respond the complex and recalcitrant structure of lignocellulosic biomass [33, 41]. In contrast, some fungi have few AA9- or AA14 LPMO-encoding genes. *Talaromyces rugulosus* contains only one AA9 LPMO- and two AA14 LPMO-encoding genes [22]. Interestingly, other species belonging to the genus of *Talaromyces* such as *T. borbonicus*, *T. marneffei*, *T. piceae*, *T. stipitatus*, and *T. verruculosus* also contain single AA9 LPMO-encoding gene in their genome in the CAZy database (http://www.cazy.org), indicating that the fungal species from this genus are poor in AA9 LPMOs. *Tr*AA14A has a dual cellulolytic/hemicellulolytic activity on natural eucalyptus pulp, indicating that it is a truly bifunctional enzyme that has evolved to simultaneously oxidize both hemicellulose and cellulose fibers in natural substrates. Thus, the bifunctional *Tr*AA14A could provide better performance to overcome the recalcitrance of the plant cell wall, compensating for fewer numbers of LPMO-encoding genes in this fungus compared to other lignocellulosic biomass-degrading fungi.

In nature, cellulolytic fungi need to produce a vast arsenal of hydrolytic and oxidative enzymes with defined substrate specificity to decompose a wide variety of recalcitrant, copolymeric plant polysaccharide structures. The vast multiplicity of fungal cellulolytic enzyme components generates considerable challenges in mining and selecting potential candidates to customize cellulase cocktails for efficient hydrolysis of biomass. LPMOs have become a crucial component in industrial cellulase cocktails for biomass hydrolysis, however, the potential of these enzymes has not been fully exploited due to the limited availability of characterized LPMOs with novel properties. In view of its unique features, *Tr*AA14A may be a potential booster in cellulolytic enzyme cocktails to efficiently break down the hemicellulose-cellulose matrix in lignocellulose [42–44]. It may also be relevant for enzymatic upgrading of heteroxylans for added-value chemicals and polymers, and the efficient hemicellulose removal for the efficient isolation of nanocellulose [45, 46].

## Conclusions

In this study, a bifunctional AA14 LPMO with dual cellulolytic/hemicellulolytic activity on natural hemi/cellulosic substrates was identified from ascomycete *T. rugulosus*. The extensive cellulose- and xyloglucan-activity together with xylan-activity on natural hemi/cellulosic substrates enables *Tr*AA14A to possess a profound boosting effect on cellulose hydrolysis. Despite there is controversy regarding the oxidative catalytic activity and functionality of AA14 LOMOs, the current studies confirmed its oxidative activity on hemi/cellulosic substrates and unveil the functional diversity of these new AA family enzymes. Clarifying the molecular determinants of the substrate specificity of *Tr*AA14A on both cellulose and hemicellulose is a fascinating subject for further studies of this AA14 LPMO.

## Methods

### Phylogenetic analysis and sequence alignment of AA14 LPMOs

The amino acid sequences of two putative AA14 LPMOs (*Tr*AA14A and *Tr*AA14B, GenBank accession no. XP035345895 and XP035350921, respectively) were retrieved from the genome of *T. rugulosus* W13939 available in the Carbohydrate-Active enZYmes database (CAZY, http://www.cazy.org/). Phylogenetic analysis was performed using various characterized AA9 LPMOs and putative AA14 LPMOs protein sequences, which were collected from CAZY database and the Joint Genome Institute portal (https://jgi.doe.gov/data-and-tools/data-systems/mycocosm/html). The sequences encoding signal peptide, CBM1 and the dCTR based on the prediction using MobiDB-lite 5.0 (https://mobidb.bio.unipd.it/) in selected genes were deleted, and only the catalytic domain was selected for phylogenetic analysis. The phylogenetic tree was constructed using the clustalW multiple sequence alignment program and then neighbor-joining method in MEGA 11.0 software (Molecular Evolutionary Genetics Analysis) [47]. Bootstrap values calculated from 1000 replicates are shown at each node. Multiple sequence alignment of *Tr*AA14A and B with the characterized *Pc*AA14A and *Pc*AA14B (GenBank ID: AUM86166.1 and AUM86167.1, respectively) was generated using MEGA (version 11) software by ClustalW program.

### Expression and purification of *Tr*AA14A

Two *Tr*AA14A- and B-encoding genes from *T. rugulosus* W13939A were synthesized by Springen (Nanjing, China) with modified codons according to the codon preference of *Pichia pastoris*. The fragments were ligated at the *Bst*BI/*Eco*RI (NEB) sites of pPICZαA expression vector (Invitrogen) to yield the expression plasmids pPICZαA-*Tr*AA14A and pPICZαA-*Tr*AA14B, in which the α-factor sequence was removed and replaced with the native signal sequences. The pPICZαA-*Tr*AA14A and pPICZαA-*Tr*AA14B were linearized using *Sac*I (NEB) and transformed into *P. pastoris* X33 by electroporation using a BIO-RAD electroporator. *Tr*AA14A was successfully expressed and the recombinant protein was purified by Ni–NTA affinity column according to the manufacture's manual. The purified *Tr*AA14A was saturated with Cu(II) before using according to the previous method [25]. In brief, the purified *Tr*AA14A (as well other AA9 LPMOs used in this study) were saturated with Cu(II) by dialysis of purified enzyme a dialysis bag (MD25-14 with 14,000 Da MWCO, Viskase, Lombard, IL, USA) in 1 mM CuSO_4_ [1000 mL in 100 mM sodium acetate (pH 5.0)] for 30 min at room temperature, then the enzyme was dialyzed in in sodium acetate buffer (pH 5.0) three times for 24 h to remove the free Cu^2+^. The purity and molecular weight of the purified *Tr*AA14A was determined using sodium dodecyl sulfate–polyacrylamide gel electrophoresis (SDS-PAGE) [12% (*w*/*v*)]. The deglycosylation of *Tr*AA14A protein was performed using Endo H (NEB) according to the manufacture’s manual. The protein concentration was determined using a BCA kit (Thermo Fisher Scientific).

### Other AA9 LPMOs and cellulolytic enzymes

The recombinant *Nc*LPMO9C, *Ep*LPMO9A, and GH5 endoglucanase (EGI) from *Neurospora crassa*, *Eupenicillium parvum* 4–14, and *Volvariella volvacea*, respectively, were produced by *P. pastoris* transformants and further purified by Ni–NTA system as described before [25, 48, 49]. The purity and molecular weights of the recombinant *Nc*LPMO9C and *Ep*LPMO9A were also checked by SDS-PAGE. CBHI from *Hypocrea jecorina*, CBHII from a microbial source, and Celluclast®1.5 L were purchased from Sigma-Aldrich, Megazyme and Novozymes, respectively. All other chemicals were of analytical grade and commercially available.

### Substrates

RAC (Regenerated amorphous cellulose, designated RAC-85) was prepared from Avicel® PH-101 (Fluka, Buchs, Switzerland, ~ 25 μm particle size, DPn ≈ 220) as described previously [50]. Mercerized fiber was prepared by incubating Avicel® PH-101 in 20% NaOH solution (*w*/*v*) with a solid to liquid ratio of 1:5 at 45 ℃ for 1 h, then the treated cellulose was neutralized with 6 M sulfuric acid to neutral pH and further washed with distilled water twice as described previously [51]. The surface morphology of mercerized fiber was analyzed by a JEOL-JSM-7600F scanning electron microscope (SEM) (JEOL, Tokyo, Japan) as described previously [52]. The size of mercerized fiber was similar as Avicel® PH-101, but the surface became rougher (Additional file 1: Fig. S7). PFI-fibrillated eucalyptus pulp (Beating revolution 5000) was prepared as previously described [52]. α and β-Cellulose were prepared from PFI-fibrillated eucalyptus pulp according to TAPPI standard technique T 203 cm-99 method (1999) [29]. Brief, PFI-fibrillated eucalyptus pulp (1.5 g) was immersed in 100 mL of 17.5% NaOH at 25 ℃ for 30 min, then diluted with 100 mL of distilled water (to final NaOH concentration of 9.45%), and continued incubated at 25 ℃ for 30 min. The residue, designated α-cellulose, was collected by filtration, then washed with distilled water to neutral pH, and freezing-dried. The supernatant after removing the residue was mixed with an equal volume of 3 N sulfuric acid and bathed in water at 80 ℃ for 10 min to allow better condensation and precipitation, and then placed at room temperature overnight. The precipitate, designated β-cellulose, was collected by centrifugation, washed to neutral pH with 25% ethanol, and dialyzed in pure water for 2 days, and then freeze-dried. The xylans, designated xylan-10% and xylan-5%, respectively, were extracted from PFI-fibrillated eucalyptus pulp with 10% and 5% aqueous NaOH solution (w/w), respectively, at 25 ℃ for 2 h. Then, the alkaline extracts were neutralized by acetic acid until pH 4.2 and stored at 4 ℃ for 12 h to precipitate the xylan and soluble low-molecular-weight cellulose chains (even some extent of decomposed celluloses). The precipitate was collected by centrifugation and dialyzed in pure water for 2 days, and then freeze dried [30]. Wheat arabinoxylan (WAX, Wheat Flour, Low Viscosity, Arabinose:Xylose = 38:62, purity > 95%), glucomannan (Konjac, Low Viscosity, Mannose:Glucose = 60:40) and xyloglucan (XG, Xylose:Glucose:Galactose:Arabinose:Other sugars = 34:45:17:2:2) from tamarind, lichenin from Icelandic Moss (β-1,3–1,4-glucan, Glucose:Arabinose:Mannose:Xylose:Galactose:Other sugars = 77.3:2.7:8.0:1.0:9.2:1.8) and beechwood xylan (BeWX, Xylose:Glucuronic Acid: Other sugars = 86.1:11.3:2.6) were purchased from Megazyme (Bray, Ireland).

### Kinetic constant analysis and H_2_O_2_ production of *Tr*AA14A and apo-*Tr*AA14A

The copper-free apo-form of *Tr*AA14A (apo-*Tr*AA14A) was prepared by dialysis of the Cu(II) saturated *Tr*AA14A in sodium acetate buffer (100 mM, pH 5.0) containing 1 mM EDTA for 12 h, then excessive EDTA was removed by dialyzing the enzyme again in sodium acetate buffer (100 mM, pH 5.0) two times for 24 h. Kinetic constants for both substrate 2,6-dimethoxyphenol (2, 6-DMP) and co-substrate H_2_O_2_ were conducted in sodium phosphate buffer (100 mM, pH 7.0) at 37 ℃ for 5 min according to previously described method [53]. For 2, 6-DMP, the concentration of H_2_O_2_ was fixed at 100 µM, and different concentrations of 2, 6-DMP (400–4000 µM) were used. For H_2_O_2_, the concentration of 2, 6-DMP was fixed at 1 mM, and different concentrations of H_2_O_2_ (50–1000 µM) were used. One unit of LPMO activity was defined as the formation of 1 µmol coerulignone (*ε*469 = 53,200 M^−1^ cm^−1^) per min under the reaction conditions. The *K*_m_ and *k*_cat_ were calculated using the Graphpad prism 7 nonlinear regression program, fitting to the Michaelis–Menten equation.

The production of H_2_O_2_ by *Tr*AA14A in the absence of cellulosic substrate was measured by a fluorimetric assay based on leucocrystal violet and horseradish peroxidase (HRP) as previously described [54]. Briefly, the reaction mixture (500 µL) containing 1 μM *Tr*AA14A and 50 µM AscA in sodium acetate buffer (50 mM, pH 5.0) was preincubated at 45 ℃ and 1000 rpm for 20, 40 and 60 min, respectively, then terminated by heating at 99 °C for 5 min. 200 µL of reaction supernatant was taken out and mixed with 25 µL of leucocrystal violet (1 mM dissolved in 0.06 M HCl) 25 µL of HRP (100 µg/mL, Sangon Biotech, Shanghai, China), and 500 μL sodium-acetate buffer (200 mM, pH 4). After mixing evenly, the color can be developed by standing for 30 s. The absorbance value was measured at 592 nm, and the concentration of H_2_O_2_ was calculated by the standard curve.

### Oxidizing activities and synergism of *Tr*AA14A on cellulosic and xyloglucan substrates

The oxidative activities of *Tr*AA14A on various cellulosic substrates including RAC-85, Avicel, mercerized fiber and α-cellulose, or xyloglucan were determined in the reaction mixture (2.0 mL) containing various substrates (5 mg), 1 μM *Tr*AA14A and 1 mM AscA in sodium acetate buffer (pH 5.0, 50 mM) in an incubator at 45 ℃ and 200 rpm for 24 h. The control reaction containing various substrates (5 mg) with AscA (1 mM), or AscA (1 mM) and Cu^2+^ (1 μM), or inactivated *Tr*AA14A (1 μM, boiled at 99 °C for 15 min) only was also performed in parallel under the same condition. To compare the cellulose-oxidizing activity of *Tr*AA14A with AA9 LPMOs, the oxidative activities on different cellulosic substrates of two previously characterized *Nc*LPMO9C and *Ep*LPMO9A were also determined under the same conditions. After the reaction, all samples were boiled at 99 ℃ for 10 min and centrifuged at 10,000 rpm for 10 min. The nonoxidized and oxidized products in the supernatant were then assayed by HPAEC-PAD [48]. Briefly, HPAEC analysis was performed on a Dionex ICS-6000 system (Dionex, Sunnyvale, CA, USA) equipped with pulsed amperometric detection (PAD) and a CarboPac PA200 analytical column (3 × 250 mm) with a CarboPac PA200 guard column (3 × 50 mm). Products were separated using 0.1 M NaOH in the mobile phase with the concentration of sodium acetate increasing from 0 to 140 mM (14 min), 140 to 300 mM (8 min), 300 to 400 mM (4 min), and then held constant at 500 mM (3 min) before re-equilibration in 0.1 M NaOH (4 min). The flow rate was set to 0.4 mL/min, the column was maintained at a temperature of 30 °C. The oxidation regioselectivity of *Tr*AA14A was determined by analyzing the products generated from RAC-85 using MALDI-TOF MS as described before [45]. In all analyses, 2,5-dihydroxybenzoic acid (DHB) in acetonitrile 30% (*v*/*v*) was used as the matrix. The synergism of *Tr*AA14A with GHs was performed in a reaction mixture (1 mL) by mixed incubation of *Tr*AA14A (1 μM) with EGI (10 μg), CBHI (20 μg), CBHII (20 μg), or Celluclast®1.5L (0.04 U), and 4 mg/mL RAC-85 or mercerized fiber, with 1 mM AscA in sodium acetate buffer (pH 5.0, 50 mM) at 45 ℃ and 1000 rpm for 1 h in a thermomixer. The control reaction in a reaction mixture (1 mL) containing individual *Tr*AA14A (1 μM), EGI (10 μg), CBHI (20 μg), CBHII (20 μg), or Celluclast®1.5L (0.04 U), and 4 mg/mL RAC-85 or mercerized fiber, with 1 mM AscA was performed under same condition. The reaction was stopped by boiling at 99 ℃ for 10 min and centrifuging at 10,000 rpm for 10 min. The reducing sugar in the supernatant was then assayed by DNS. The degree of synergy (DS) of the coupled enzyme mixtures was calculated by Eq. (1):

1 $$ {\text{DS}} = {\text{RS}}_{({\text{GH}} + Tr{\text{AA14A}})} /\left( {{\text{RS}}_{{\text{GH}}} + {\text{ RS}}_{Tr{\text{AA14A}}} } \right) $$

where RS_(GH+ *Tr*AA14A)_ is the reducing sugar released from the enzyme mixture of GH and *Tr*AA14A, and (RS_GH_ + RS_*Tr*AA14A_) is the sum of the reducing sugar released from the single GH enzyme and *Tr*AA14A.

### Oxidizing activities of *Tr*AA14A on hemi/cellulosic substrates

Various commercial or self-prepared hemi/cellulosic substrates including Wheat arabinoxylan (WAX), glucomannan, lichenin, beechwood xylan, PFI-fibrillated eucalyptus pulp, β-cellulose, xylan-10%, and xylan-5% were used for assessing the oxidative activity of *Tr*AA14A in this study. For these substrates, the reaction was performed in the reaction mixture (500 μL) containing 5 mg substrate, 1 μM *Tr*AA14A and 1 mM AscA in sodium acetate buffer (pH 5.0, 50 mM) in a thermomixer at 45 ℃ and 1000 rpm for 1 h. The control reaction containing various substrates (5 mg) with AscA (1 mM), or AscA (1 mM) and Cu^2+^ (1 μM), or inactivated *Tr*AA14A (1 μM, boiled at 99 °C for 15 min) only was also performed in parallel under the same condition. After reaction, all samples were immediately filtered by passing the reaction mixture through a membrane with a pore size of 0.22 μm and centrifuged at 10,000 rpm for 10 min. The nonoxidized and oxidized products in the supernatants were then assayed by HPAEC-PAD. The profiles of nonoxidized and oxidized products by *Tr*AA14A were determined by analyzing the products generated from PFI-fibrillated eucalyptus pulp and xyloglucan using MALDI-TOF MS. The chemical composition analysis of PFI-fibrillated eucalyptus pulp was carried out according to the analytical procedure provided by the National Renewable Energy Laboratory (NREL/TP-510-42618) standard method [55]. The neutral carbohydrates in the supernatant were then quantified by HPLC (Agilent 1260, refractive index detector G1362A, USA) with Bio-Rad Aminex HPX-87H column (300 mm × 7.8 mm, USA) which was operated at 55 °C with 5 mM H_2_SO_4_ as the mobile phase at the flow rate of 0.6 mL/min as previously described by Chen et al. [56], while 4-o-methyl glucuronic acid (MeGlcA) and glucuronic acid (GlcA) were quantified by HPAEC-PAD.

### Structure modelling of *Tr*AA14A

The three-dimensional (3D) protein structural model of *Tr*AA14A was firstly performed using the AlphaFold2 servers (https://colab.research.google.com/github/sokrypton/ColabFold/blob/main/AlphaFold2.ipynb), and SWISS-MODEL servers (https://swissmodel.expasy.org/interactive) uploading the primary sequence barring the signal peptide via default parameter. Then the output simulated structural model quality was evaluated via QMEAN4 and QMEANDisco (https://swissmodel.expasy.org/qmean/), to ensure that the modeling servers returned a reliable model for *Tr*AA14A. The constructed model of *Tr*AA14A was also superposed with *Pc*AA14B (PDB code 5NO7) and C1-oxidizing AA9 LPMO *Nc*LPMO9F (PDB code 4QI8) by uploading the PDB format files to the mTM-align server (http://yanglab.nankai.edu.cn/mTM-align/index.html), and the copper position was selected based on structural alignment with the template *Nc*LPMO9F. The cartoon representation of the LPMOs tridimensional models and the stick representation of active site residues were prepared using PyMOL 2.5. For the simulation of electrostatic potentials of the *Tr*AA14A, *Pc*AA14B, and *Nc*LPMO9F, the protonation states were assigned to each atom of the structures at pH 5.0 and pH 6.0 according to the parameters from the AMBER force field by PROPKA algorithm used by PDB2PQR web server [57]. And the electrostatic potentials were then visualized using the PyMOL 2.5.7 with Adaptive Poisson-Boltzmann Software package (APBS) plugin [58].

### Statistical analysis

All described experiments were performed in triplicate and all the reported data are means of the three samples. In addition, the Student’s *t*-test analysis was used to determine the statistical difference between the two groups, when *p* < 0.05, the data difference was statistically significant.

### Supplementary Information

**Additional file 1: Figure S1.** Multiple-sequence alignment of AA14 LPMOs and xylan-active AA9 LPMOs. The amino acid residues forming the His brace are indicated as the solid red arrow. The specific loop regions conducive to shaping the substrate-binding surface are indicated by labeled black lines according to the L2, L3, LS, and LC loops of *Nc*LPMO9F. **Figure S2.** **A** SDS-PAGE of purified *Tr*AA14A and deglycosylated *Tr*AA14A. M, protein marker; Line1, purified *Tr*AA14A; Line2, deglycosylated *Tr*AA14A. **B** SDS-PAGE analysis of the purity and molecular weights of LPMOs. **Figure S3.** Figure S3. Kinetics curves for the peroxidase reaction rate and H_2_O_2_ production by *Tr*AA14A and apo-*Tr*AA14A. **A** The kinetics curve for H_2_O_2_. **B** The kinetics curve for 2,6-DMP. **C** H_2_O_2_ production. **Figure S4.** HPAEC-PAD analysis of reaction products generated by *Tr*AA14A from Avicel, mercerized fiber, α-cellulose and WAX. **Figure S5.** HPAEC-PAD analysis of reaction products generated by *Nc*LPMO9C and *Ep*LPMO9A from RAC-85 and Avicel. **A**, **B** The reaction products generated from RAC-85 by *Nc*LPMO9C and *Ep*LPMO9A, respectively.* C*, **D** The reaction products generated from Avicel by* Nc*LPMO9C and *Ep*LPMO9A, respectively. **Figure S6.** HPAEC-PAD analysis of reaction products generated by *Tr*AA14A on various and hemi/cellulosic substrates. **Figure S7.** SEM microscopy of mercerized fiber prepared from Avicel® PH-101 with different magnification. **Table S1.** The contents of neutral sugars and uronic acids of polysaccharides in different substrates.

## Data Availability

All data generated or analyzed during this study are included in this published article and its Additional information files.

## Abbreviations

AA14: Auxiliary activity family 14
LPMO: Lytic polysaccharide monooxygenase
GH: Glycoside hydrolase family
RAC: Regenerated amorphous cellulose
XG: Xyloglucan
2: 6-DMP, 2,6-dimethoxyphenol
POD: Horseradish peroxidase
CBAX: Corn bran arabinoxylan
WAX: Wheat arabinoxylan
AscA: Ascorbic acid
DNS: 3,5-Dinitrosalicylic acid
HPAEC-PAD: High performance anion exchange chromatography equipped with pulsed amperometric detection
HPLC: High performance liquid chromatography
MALDI-TOF MS: Matrix-assisted laser desorption/ionization-time-of-fight mass spectrometry
