# Supplementary material for: A novel AA14 LPMO from Talaromyces rugulosus with bifunctional cellulolytic/hemicellulolytic activity boosted cellulose hydrolysis
Source: Biotechnol Biofuels Bioprod. 2024 Feb 23;17:30. doi: 10.1186/s13068-024-02474-9 (PMC10885436; doi:10.1186/s13068-024-02474-9)
Supplement: Supplementary file 1 — Additional file 1: Figure S1. Multiple-sequence alignment of AA14 LPMOs and xylan-active AA9 LPMOs. The amino acid residues forming the His brace are indicated as the solid red arrow. The specific loop regions conducive to shaping the substrate-binding surface are indicated by labeled black lines according to the L2, L3, LS, and LC loops of NcLPMO9F. Figure S2. A SDS-PAGE of purified TrAA14A and deglycosylated TrAA14A. M, protein marker; Line1, purified TrAA14A; Line2, deglycosylated TrAA14A. B SDS-PAGE analysis of the purity and molecular weights of LPMOs. Figure S3. Figure S3. Kinetics curves for the peroxidase reaction rate and H2O2 production by TrAA14A and apo-TrAA14A. A The kinetics curve for H2O2. B The kinetics curve for 2,6-DMP. C H2O2 production. Figure S4. HPAEC-PAD analysis of reaction products generated by TrAA14A from Avicel, mercerized fiber, α-cellulose and WAX. Figure S5. HPAEC-PAD analysis of reaction products generated by NcLPMO9C and EpLPMO9A from RAC-85 and Avicel. A, B The reaction products generated from RAC-85 by NcLPMO9C and EpLPMO9A, respectively. C, D The reaction products generated from Avicel by NcLPMO9C and EpLPMO9A, respectively. Figure S6. HPAEC-PAD analysis of reaction products generated by TrAA14A on various and hemi/cellulosic substrates. Figure S7. SEM microscopy of mercerized fiber prepared from Avicel® PH-101 with different magnification. Table S1. The contents of neutral sugars and uronic acids of polysaccharides in different substrates. [file 13068_2024_2474_MOESM1_ESM.docx]

Additional file

A novel AA14 LPMO from *Talaromyces rugulosus* with bifunctional cellulolytic/hemicellulolytic activity boosted cellulose hydrolysis

Kaixiang Chen, Xu Zhao, Peiyu Zhang, Liangkun Long and Shaojun Ding*

The Co-Innovation Center of Efficient Processing and Utilization of Forest Resources, Jiangsu Key Lab for the Chemistry & Utilization of Agricultural and Forest Biomass, College of Chemical Engineering, Nanjing Forestry University, Nanjing 210037 Jiangsu, China.

*To whom correspondence should be addressed: Shaojun Ding (E-mail: dshaojun@njfu.edu.cn; Telephone: +86 85427544)


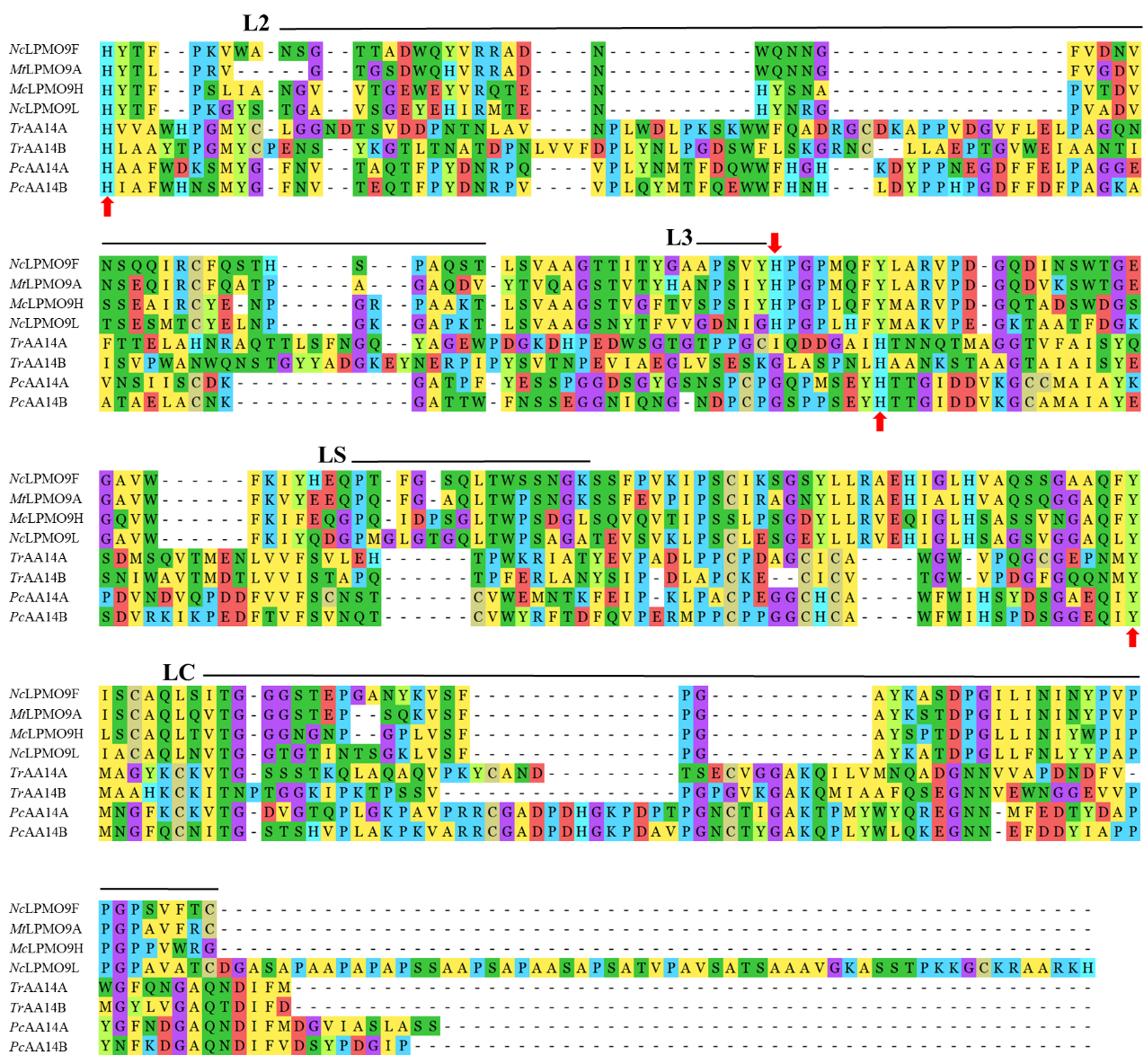


Figure S1. Multiple-sequence alignment of AA14 LPMOs and xylan-active AA9 LPMOs. The amino acid residues forming the His brace are indicated as the solid red arrow. The specific loop regions conducive to shaping the substrate-binding surface are indicated by labeled black lines according to the L2, L3, LS, and LC loops of *Nc*LPMO9F.


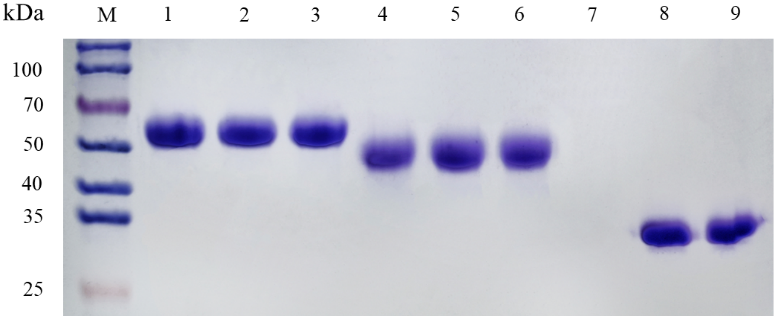

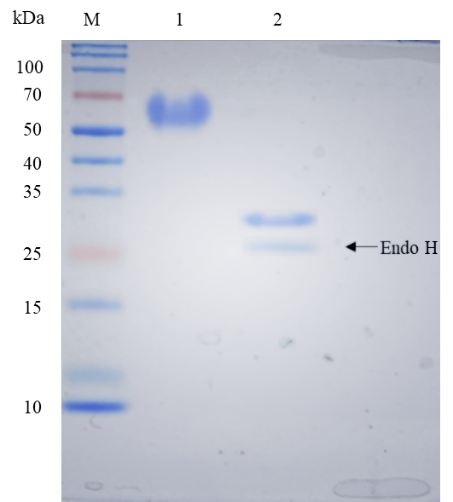


A

B

Figure S2. (A) SDS-PAGE of purified *Tr*AA14A and deglycosylated *Tr*AA14A. M, protein marker; Line1, purified *Tr*AA14A; Line2, deglycosylated *Tr*AA14A. (B) SDS-PAGE analysis of the purity and molecular weights of LPMOs. Lane M, molecular weight marker; lanes 1- 3, heat-inactivated *Tr*AA14A (boiled at 99 °C for 15 min), apo-*Tr*AA14A and *Tr*AA14A, respectively; 4-6: heat-inactivated *Nc*LPMO9C (boiled at 99 °C for 15 min), apo-*Nc*LPMO9C and *Nc*LPMO9C, respectively; 7-9: heat-inactivated *Ep*LPMO9A (boiled at 99 °C for 15 min), apo-*Ep*LPMO9A, and *Ep*LPMO9A, respectively. For heat-inactivated LPMOs, after boiled at 99 °C for 15 min, the samples were centrifugated at 10,000 rpm for 10 min, and then 50 μL of supernatant was loaded onto SDS-PAGE to detect the content of soluble protein in the supernatant.


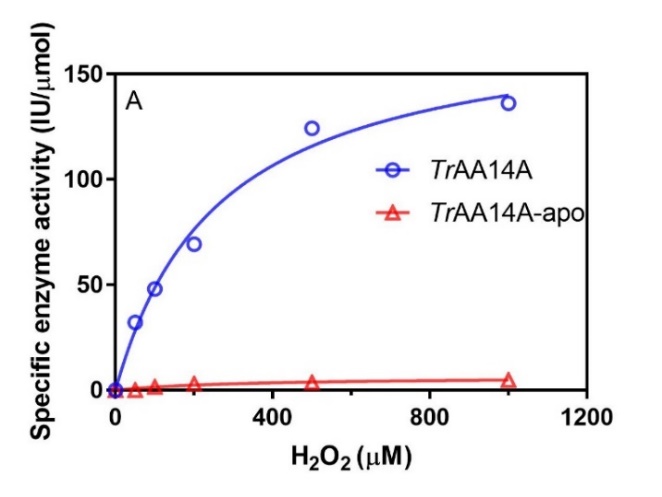

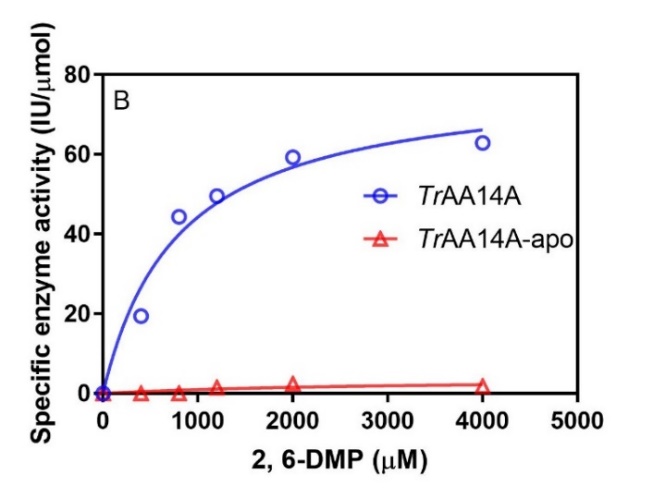

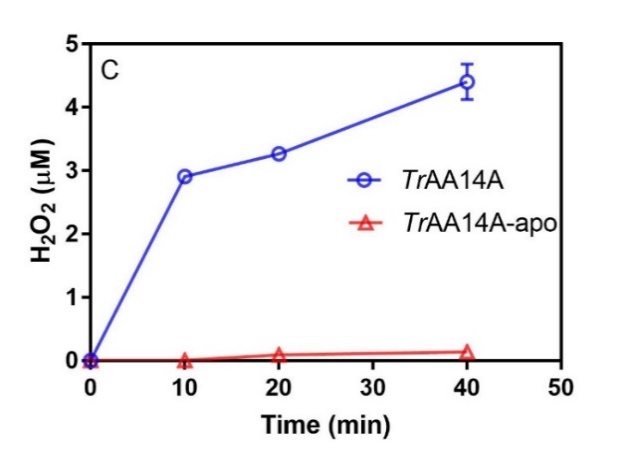


Figure S3. Kinetics curves for the peroxidase reaction rate and H_2_O_2_ production by *Tr*AA14A and apo-*Tr*AA14A. (A) The kinetics curve for H_2_O_2_. (B) The kinetics curve for 2,6-DMP. (C) H_2_O_2_ production.


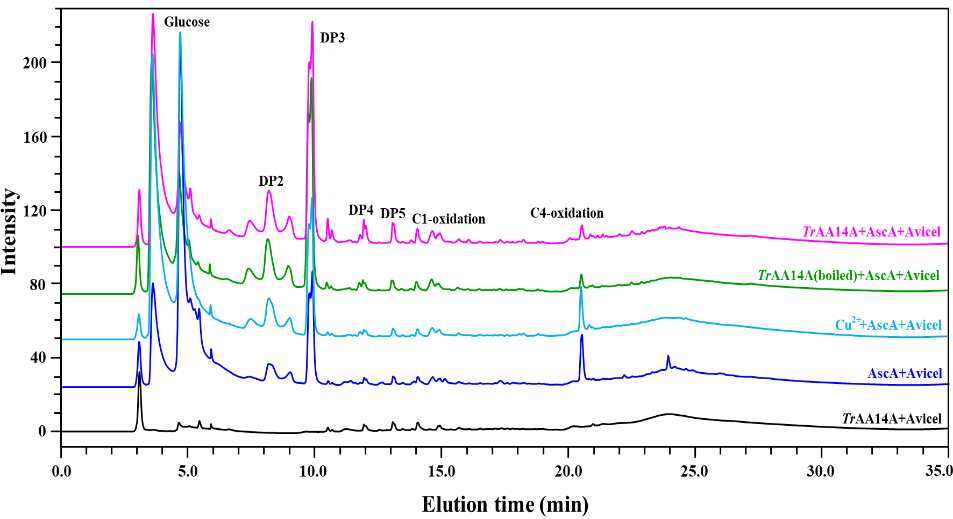


A


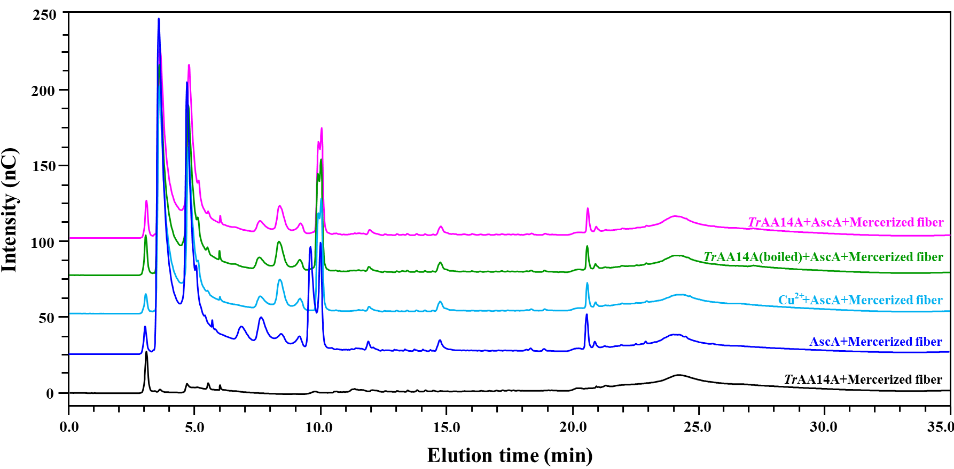


B


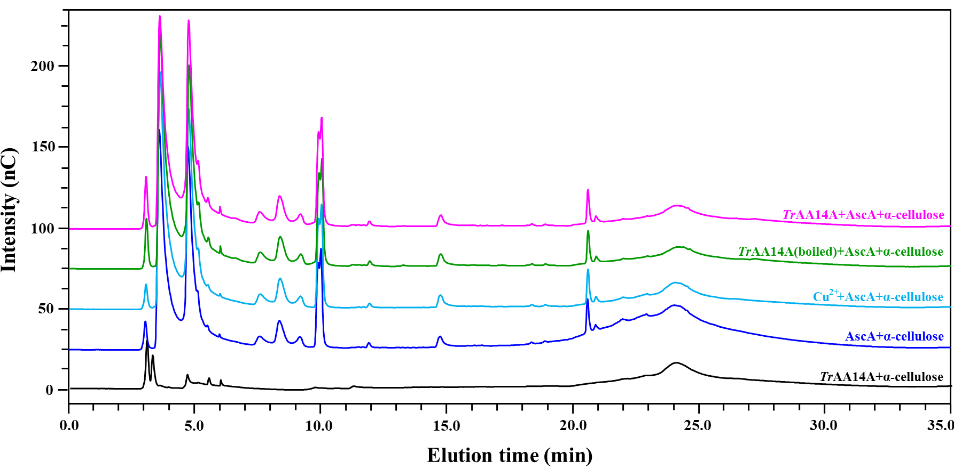


C


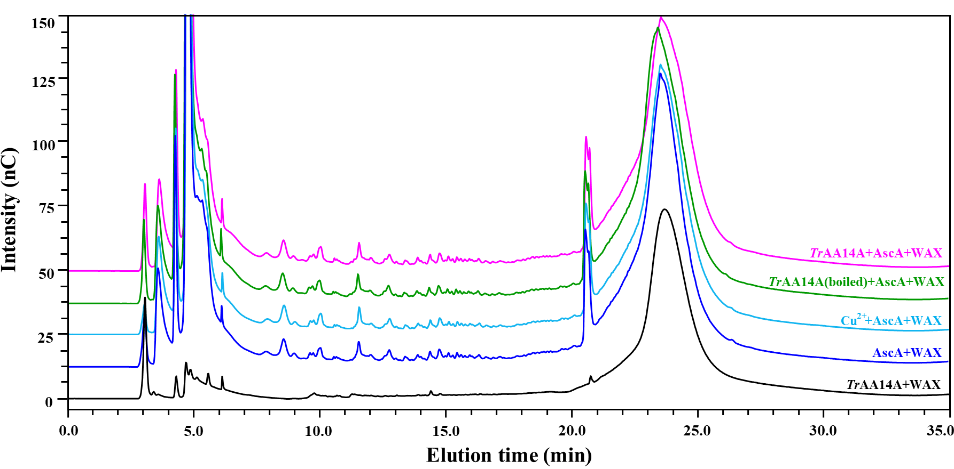


D

Figure S4. HPAEC-PAD analysis of reaction products generated by *Tr*AA14A from Avicel, mercerized fiber, α-cellulose and WAX. (A) The reaction products generated from mercerized fiber. (B) The reaction products generated from α-cellulose. (C) The reaction products generated from WAX. The oxidative activities of *Tr*AA14A towards cellulosic substrates were determined in the reaction mixture (2.0 mL) containing various substrates (5 mg), 1 μM *Tr*AA14A and 1 mM AscA in sodium acetate buffer (pH 5.0, 50 mM) in an incubator at 45 ℃ and 200 rpm for 24 h. The control reaction containing various substrates (5 mg) with AscA (1 mM), or AscA (1 mM) and Cu^2+^ (1 μM), or heat-inactivated *Tr*AA14A (designated *Tr*AA14A(boiled), boiled at 99 °C for 15 min) (1 μM) and AscA (1 mM) was also performed in parallel under the same condition. The oxidative activities of *Tr*AA14A towards WAX was performed in the reaction mixture (500 μL) containing 5 mg substrate, *Tr*AA14A (1 μM) and AscA (1 mM) in sodium acetate buffer (pH 5.0, 50 mM) in a thermomixer at 45 ℃ and 1000 rpm for 1 h. The control reaction containing various substrates (5 mg) with AscA (1 mM), or AscA (1 mM) and Cu^2+^ (1 μM), or heat-inactivated *Tr*AA14A (designated *Tr*AA14A(boiled), boiled at 99 °C for 15 min) (1 μM) and AscA (1 mM) was also performed in parallel under the same condition. After reaction, the reaction solution was immediately filtered by passing the reaction mixture through a membrane with a pore size of 0.22 μm and analyzed by HPAEC-PAD.


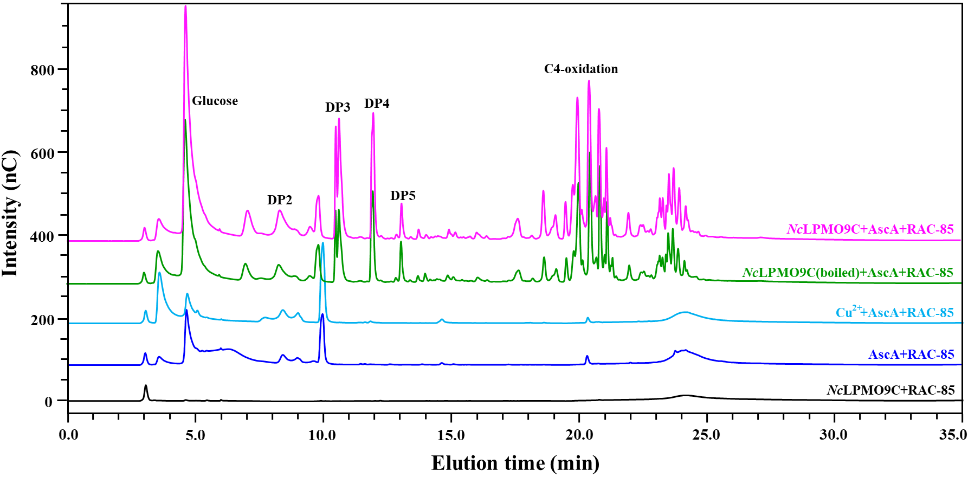


A


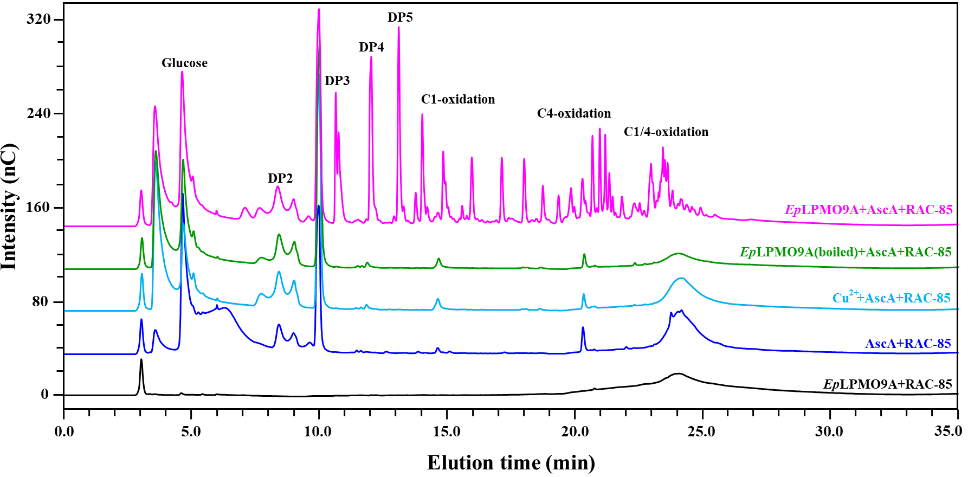


B


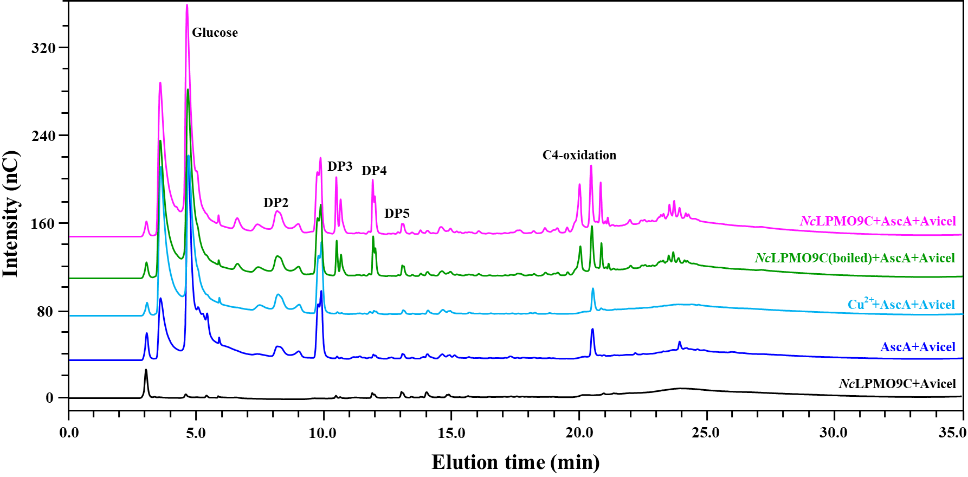


C


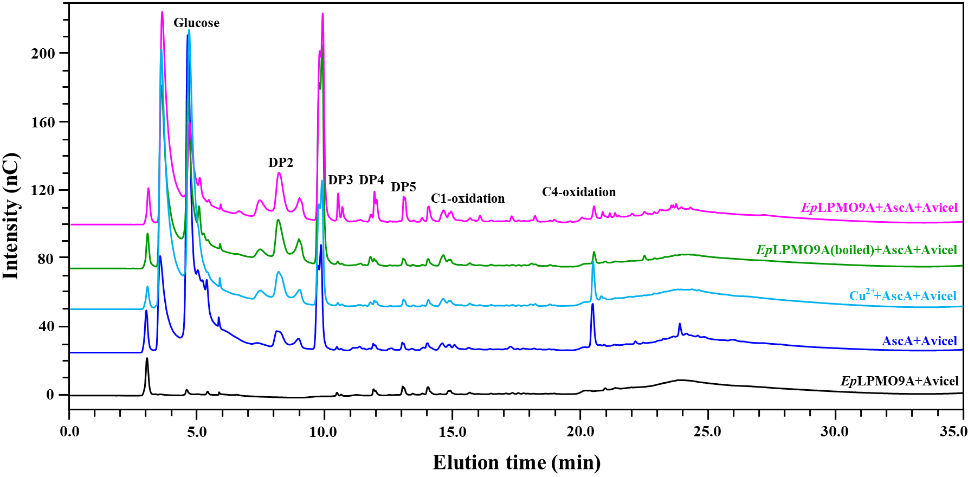


D

Figure S5. HPAEC-PAD analysis of reaction products generated by *Nc*LPMO9C and *Ep*LPMO9A from RAC-85 and Avicel. (A) (B) The reaction products generated from RAC-85 by *Nc*LPMO9C and *Ep*LPMO9A, respectively. (C) (D) The reaction products generated from Avicel by *Nc*LPMO9C and *Ep*LPMO9A, respectively. The oxidative reaction of AA9 LPMO towards cellulosic substrates were performed in the reaction mixture (2.0 mL) containing various substrates (5 mg), 1 μM AA9 LPMO and 1 mM AscA in sodium acetate buffer (pH 5.0, 50 mM) in an incubator at 45 ℃ and 200 rpm for 24 h. The control reaction containing various substrates (5 mg) with AscA (1 mM), or AscA (1 mM) and Cu^2+^ (1 μM), or heat-inactivated AA9 LPMO (1 μM, designated *Nc*LPMO9C and *Ep*LPMO9A, boiled at 99 °C for 30 min and 15 min, respectively) only was also performed in the same condition.


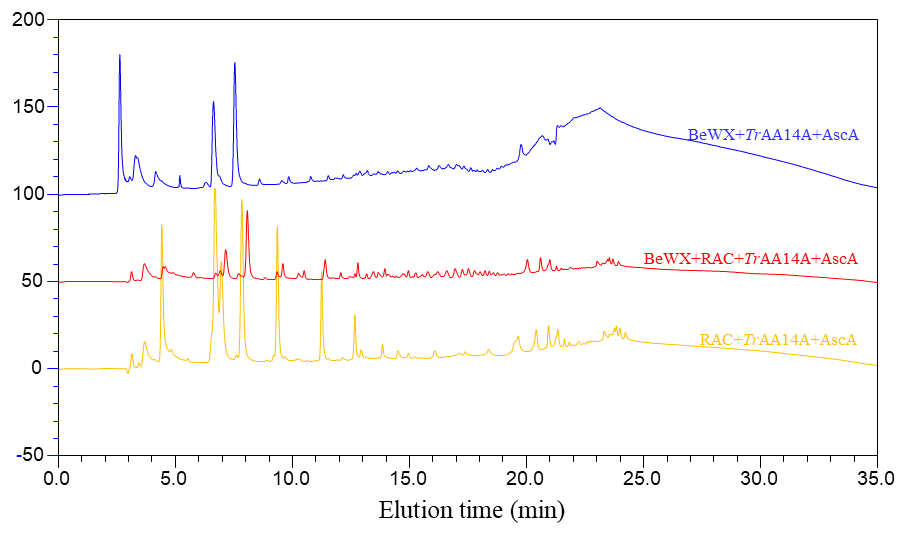
 Figure S6. HPAEC-PAD analysis of reaction products generated by *Tr*AA14A on various and hemi/cellulosic substrates. The reaction was performed in the reaction mixture (500 μL) containing 5 mg WAX, *Tr*AA14A (1 μM) and AscA (1 mM) in sodium acetate buffer (pH 5.0, 50 mM) in a thermomixer at 45 ℃ and 1000 rpm for 1 h. The control reaction containing WAX (5 mg) with AscA (1 mM), or *Tr*AA14A only was also performed in parallel under the same condition.


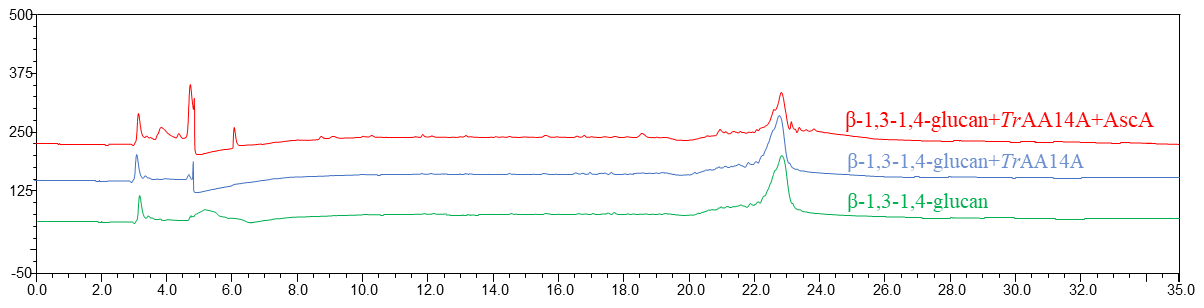

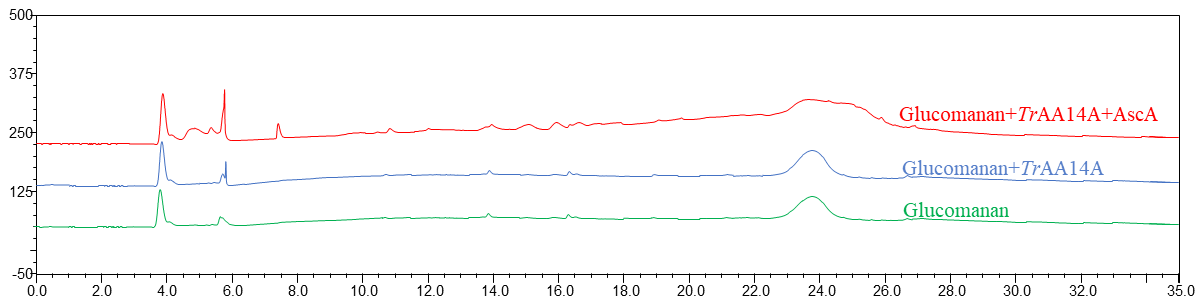

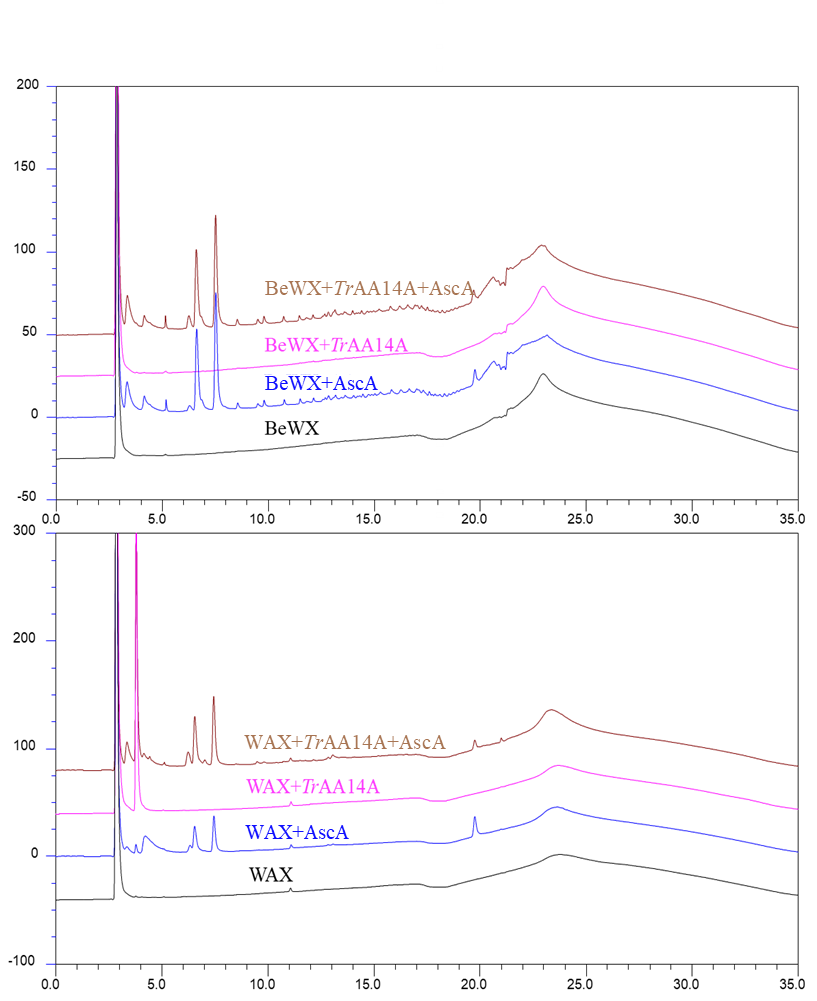


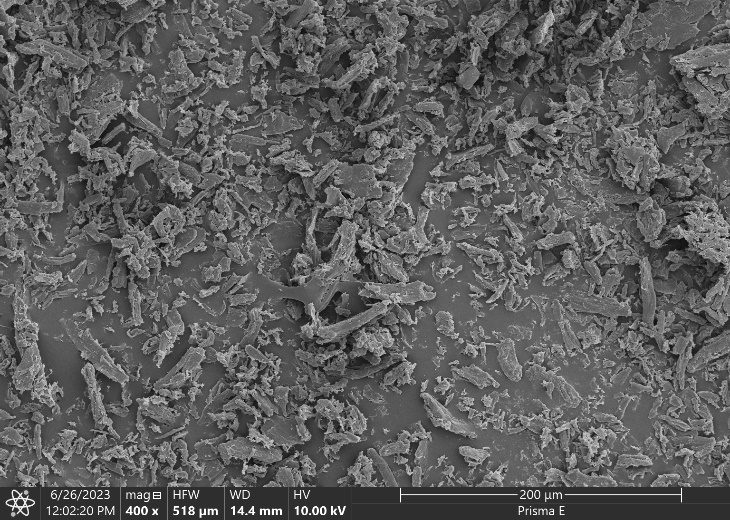


A


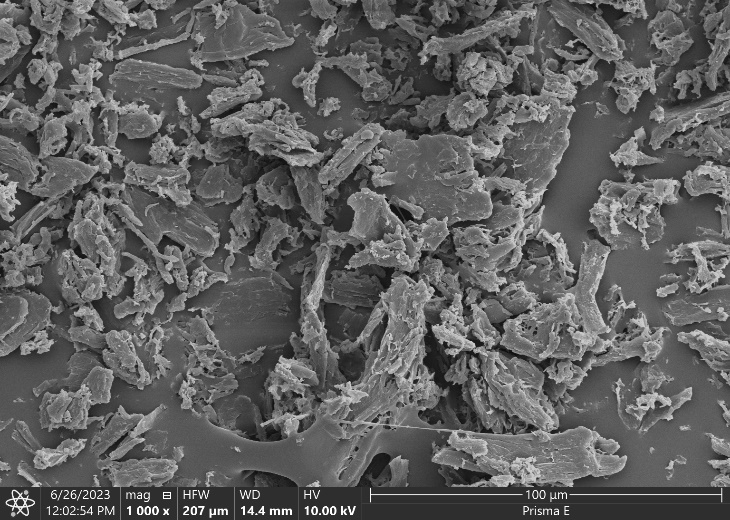


B


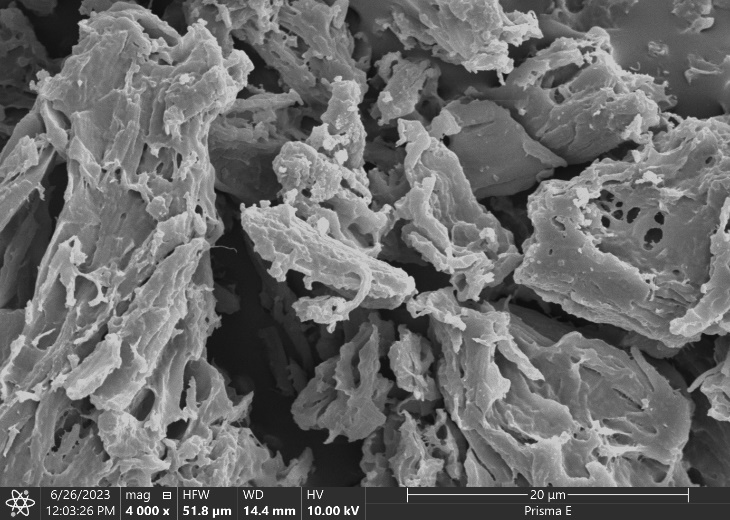


C

Figure S7. SEM microscopy of mercerized fiber prepared from Avicel® PH-101 with different magnification.

Table S1. The contents of neutral sugars and uronic acids of polysaccharides in different substrates

| Sample | MeGlcA (%) | | GlcA (%) | | Glucose (%) | | Xylose (%) | Arabinose (%) |
| --- | --- | --- | --- | --- | --- | --- | --- | --- |
| Eucalyptus pulp | | 0.464 | | 0.079 | | 67.46 | 21.57 | \ |
| Xylan-5% | | 1.071 | | 0.355 | | 1.39 | 84.00 | \ |
| Xylan-10% | | 0.757 | | 0.373 | | 44.13 | 59.55 | \ |
| β-Cellulose | | 0.792 | | 0.473 | | 44.40 | 47.50 | \ |
| α-Cellulose | | ND | | ND | | 99.31 | 3.31 | ND |

ND: Not detected
